# Assembly and Analysis of Unmapped Genome Sequence Reads Reveal Novel Sequence and Variation in Dogs

**DOI:** 10.1038/s41598-018-29190-3

**Published:** 2018-07-18

**Authors:** Lindsay A. Holden, Meharji Arumilli, Marjo K. Hytönen, Sruthi Hundi, Jarkko Salojärvi, Kim H. Brown, Hannes Lohi

**Affiliations:** 1 Department of Biology, Portland State University, Portland, Oregon, USA; 2 Research Programs Unit, Molecular Neurology, University of Helsinki, Helsinki, Finland; 3 Department of Veterinary Biosciences, University of Helsinki, Helsinki, Finland; 4 Folkhälsan Institute of Genetics, Helsinki, Finland; 5 Research Programme on Individuals and Populations, Faculty of Biological and Environmental Sciences, University of Helsinki, Helsinki, Finland; 6 School of Biological Sciences, Nanyang Technological University, Singapore, Singapore

## Abstract

Dogs are excellent animal models for human disease. They have extensive veterinary histories, pedigrees, and a unique genetic system due to breeding practices. Despite these advantages, one factor limiting their usefulness is the canine genome reference (CGR) which was assembled using a single purebred Boxer. Although a common practice, this results in many high-quality reads remaining unmapped. To address this whole-genome sequence data from three breeds, Border Collie (n = 26), Bearded Collie (n = 7), and Entlebucher Sennenhund (n = 8), were analyzed to identify novel, non-CGR genomic contigs using the previously validated pseudo-*de novo* assembly pipeline. We identified 256,957 novel contigs and paired-end relationships together with BLAT scores provided 126,555 (49%) high-quality contigs with genomic coordinates containing 4.6 Mb of novel sequence absent from the CGR. These contigs close 12,503 known gaps, including 2.4 Mb containing partially missing sequences for 11.5% of Ensembl, 16.4% of RefSeq and 12.2% of canFam3.1+ CGR annotated genes and 1,748 unmapped contigs containing 2,366 novel gene variants. Examples for six disease-associated genes (*SCARF2*, *RD3*, *COL9A3*, *FAM161A*, *RASGRP1 and DLX6)* containing gaps or alternate splice variants missing from the CGR are also presented. These findings from non-reference breeds support the need for improvement of the current Boxer-only CGR to avoid missing important biological information. The inclusion of the missing gene sequences into the CGR will facilitate identification of putative disease mutations across diverse breeds and phenotypes.

## Introduction

Domesticated dog breeds show extreme phenotypic diversity in traits such as size (Great Dane vs. Chihuahua), morphology (Dachshund vs. Saluki), coat color (Maltese vs. Black Lab), coat type (Dalmatian vs. Border Collie) and behavior (herding vs. hunting). This diversity is due primarily to the relatively short history of extreme selective breeding for specific traits of interest^1^. The typically small number of individuals used for founding the individual breeds has resulted in substantial genetic bottlenecks and intense selection. A negative side-effect is an increased presence of deleterious or negative alleles (i.e., disease-related genes) through linkage with desired physical or behavioral traits^2^.

Purebred dogs are excellent large animal models for studying genetic studies due to their well-documented pedigrees, thorough veterinary pathology, and a high level of linkage disequilibrium (LD) in the dog genome which varies across chromosomes and breeds^3,4^. These factors facilitate genomic association studies in dogs because large LD requires fewer individuals and markers to identify genomic loci associated with phenotypic traits or diseases^5^. There are currently over 700 catalogued heritable diseases in dogs of which over 200 have been characterized at the molecular level (Online Mendelian Inheritance in Animals, OMIA) (Sargan 2004).

Our current understanding of traits in dogs is reliant upon the quality of the canine genome reference (CGR). The current CGR was created with sequences generated from a single female boxer named Tasha^6^. Using a single individual for *de novo* assembly of a reference genome is a common practice, but disregards existing variation within and among individual Boxers—in the case of the CGR—and, more importantly, between breeds that number in the hundreds. The absence of overall canid genetic diversity in the CGR is evident from microsatellite and SNP data which indicate that variation between dog breeds accounts for roughly 27% of total genetic variation as compared to 5–10% genetic differentiation in humans^7^. Given this information, it is likely that a considerable amount of existing genetic diversity within and among purebred domestic dogs is not represented in the CGR. We have investigated the extent of this limitation by analyzing the whole genome sequence data from three non-reference breeds—Border Collie, Bearded Collie, and Entlebucher Sennenhund—to uncover undescribed genetic content that exists outside of the CGR using a pseudo-*de novo* assembly of unmappable paired-end reads.

## Results

### *Pseudo-*de novo *Assembly*

**Figure 1:**
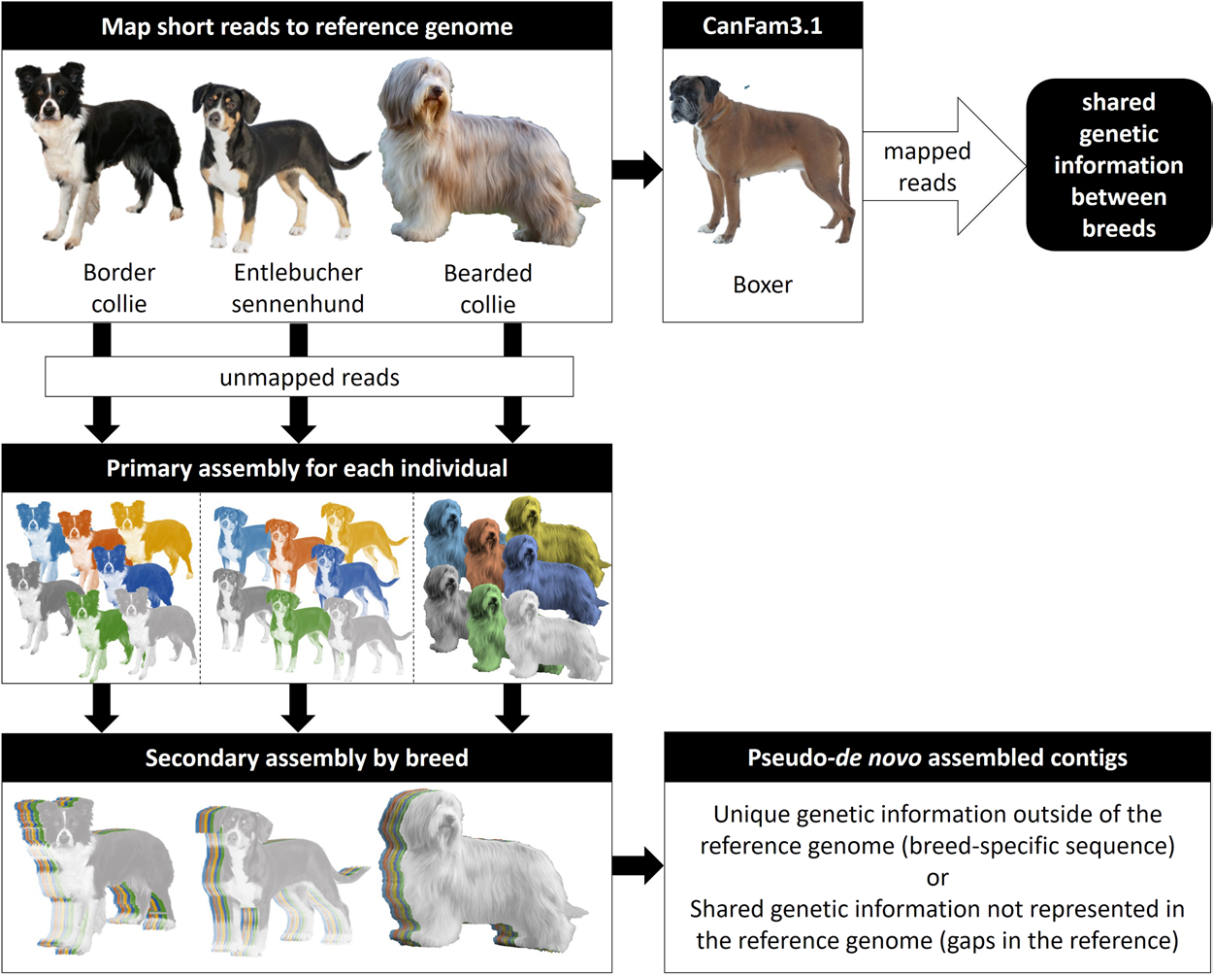
Infographic representation of the *de novo* assembly across the three breeds. Unmapped reads from each breed were assembled into primary assembly for each individual and pooled by breed to generate secondary assemblies. Boxer reads were mapped to the secondary assemblies to determine novel and shared genetic information across the assemblies. Images for Entlebucher Sennenhund, Bearded Collie and Boxer are obtained from Anne Teijula, Lotta Paakkanen and Outi Toni respectively.

Alignments of cleaned sequence data from Border Collie (n = 26), Bearded Collie (n = 7), and Entlebucher Sennenhund (n = 8) breeds to the RefSeq CGR (CanFam3.1; GCF_000002285.3) resulted in 8.6%, 4.12%, and 7.98% of unmapped reads per breed, respectively. Primary pseudo-*de novo* assembly of individuals produced an average of 654,517, 120,393, and 174,309 contigs covering 220 Mb, 47.4 Mb, and 59.5 Mb with an average N50 of 308, 478, and 386 for Border Collies, Bearded Collies, and Entlebucher Sennenhunds, respectively (Table 1). Secondary assemblies were built by pooling all individuals per breed and produced 256,957, 7476, and 15,455 contigs covering 312 Mb, 15.9 Mb, and 23.2 Mb in Border Collie, Bearded Collie, and Entlebucher Sennenhund breeds, respectively (Fig. 1). The secondary assemblies per breed have an N50 of 1374, 3174, and 2156 bp with a maximum contig length of 32 kb for Border Collies, 27 kb for Bearded Collies, and 20 kb for Entlebucher Sennenhunds (Supplemental Fig. S1**)** and represent contigs that are shared within each breed.

**Table 1:** Primary and secondary data.

Breed	Assembly	Contigs	Consensus (bp)	Largest Contig (bp)	N50
Border Collie	Primary (avg)	654,517	220,364,358	16,305	308
	Secondary	256,957	312,472,893	32,04	1,374
Bearded Collie	Primary (avg)	120,393	47,478,915	62,905	478
	Secondary	7,476	15,967,624	27,27	3,147
Entlebucher Sennenhund	Primary (avg)	174,309	59,493,588	16,863	386
	Secondary	15,455	23,200,436	20,32	2,156

The unaligned reads in the three breeds were compiled into primary (per individual) contig assemblies using MIRA 4.0 which were combined and assembled into secondary assemblies.

**Figure 2:**
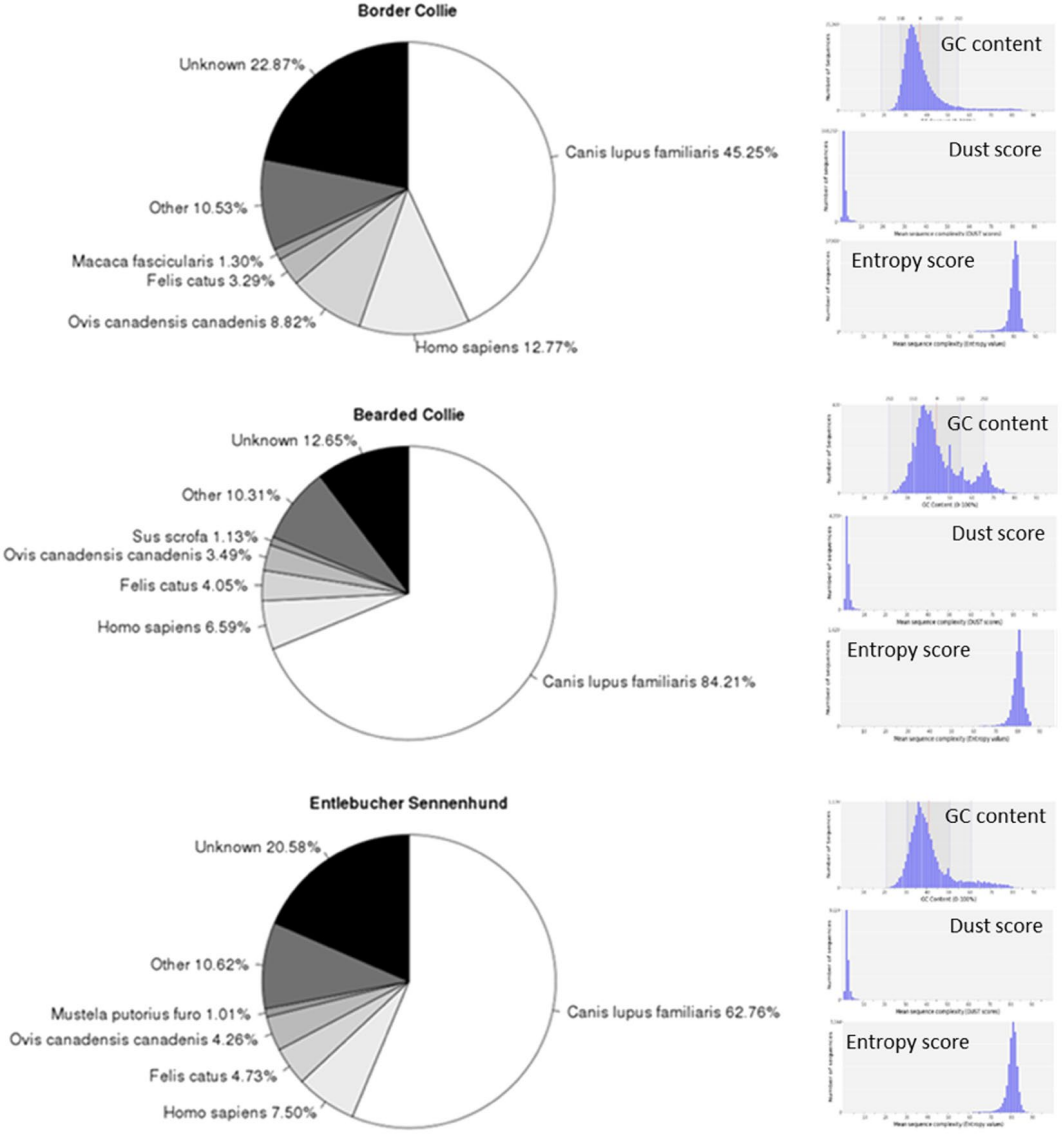
BLASTn hits for contigs from 3 domestic dog breeds. We queried all *de novo* contigs from Border Collie, Bearded Collie, and Entlebucher Sennenhund using BLASTn for nucleotide sequence similarity against the full NCBI nr/nt non-redundant nucleotide database. Single top-hit results are binned by species or identified as not aligning (unknown) to the nr/nt database. To the right of BLASTn pie charts are GC content and complexity scores of secondary *de novo* contig assemblies. GC content, DUST complexity score, and Entropy complexity scores for Border Collie, Bearded Collie, and Entlebucher Sennenhund contigs. For reference, GC content of CanFam3.1 is 41.3%. Low DUST scores (<7) and high Entropy scores (>70) indicate high complexity (fewer short repeats), as seen in all three breeds.

Mean GC content of secondary assembly contigs is in agreement with the GC content of the current CGR (canFam3.1): 37.59 ± 9.69% for Border Collies, 44.80 ± 11.17% for Bearded Collies, and 41.14 ± 10.44% for Entlebucher Sennenhunds (Fig. 2). Contigs from all three breeds had mean DUST complexity scores of <4 and mean Entropy complexity scores >80, confirming the non-redundancy and quality of breed-specific contigs. The secondary assembly was realigned to the GenBank assembly (GCA_000002285.2) using BLASTn, with the aim of incorporating all publicly available genomic variation and identifying only novel sequences within the secondary assembly. This refinement revealed that 15.85% (40,717) of the contigs in Border Collie, 12.14% (907) of the contigs in Bearded Collie, and 4.3% (676) of Entlebucher Sennenhund contigs already exist in the GenBank assembly with 100% sequence identity and 100% coverage. This refinement resulted in 216,240 unmapped contigs (85.15%) for Border Collie, 6,569 (87.86%) for Bearded Collie, and 14,779 (95.7%) for the Entlebucher Sennenhund not found in the CGR. Sanger sequence reads from the original canFam1.0 assembly based on an individual Boxer were obtained and mapped to the CGR. 14.7% of the Boxer reads (6473191 out of 43832662) did not map to the reference. Of the unmapped Boxer reads, 98.61% (6383295), 99.37% (6432270), and 99.27% (6473191) reads did not align to Border Collie, Bearded Collie, and Entlebucher Sennenhund secondary assemblies, respectively. Boxer Sanger reads with high quality alignments (MQ >  = 30) aligned to 2.3% of Border Collie contigs, 27.8% of Bearded Collie contigs, and 17.6% of Entlebucher Sennenhund contigs. The very small number of Boxer reads aligning to the breed-specific secondary assembly contigs indicates that the majority of the pseudo-*de novo* assembled contigs represent breed-specific variation and are not due to errors or gaps in the reference assembly.

### Sequence similarity and PCR Validation

BLASTn of *de novo* Border Collie contigs against the complete NCBI nr/nt database produced 166,785 contigs (77.12%) aligning to 33,923 unique predicted and confirmed genes across 356 species (Fig. 2). Top hits for Border Collie contigs partitioned to 45.25% dog, 12.77% human, 8.82% bighorn sheep, 3.29% cat, and 10.53% other species, while 22.87% of sequences did not have any BLAST alignment. BLASTn of *de novo* Bearded Collie contigs produced 5,738 contigs (87.34%) aligning to 1,711 unique predicted and confirmed genes across 99 species. Similarly, BLASTn of *de novo* Entlebucher Sennenhund contigs produced 11,737 contigs (79.41%) aligning to 3,247 unique predicted and confirmed genes across 130 species. Taxonomic profile of BLASTn hits from all the breeds are primarily assigned to species of the orders of carnivora, primate, and rodenta and suggests that secondary assemblies are free from bacterial sequence contamination.

**Figure 3:**
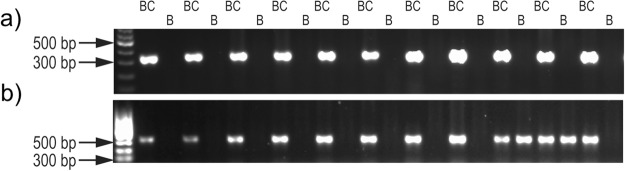
Polymerase chain reaction (PCR) examples of the contigs. (**a**) Cropped gel image showing the amplification of the contig bc_c107501 in all Border Collies (BC) and no Boxers (B). (**b**) Cropped gel image showing variable amplification of the contig bc_c222950 in Border Collies and Boxers. Full-length gels are presented in Supplementary Fig. S2.

Six contigs representing protein coding sequences were chosen for PCR amplification in an additional 94 samples: 47 each from Border Collies and Boxers. One of the contigs, bc_c107501, was found exclusively in Border Collies (Fig. 3a) while contig bc_c222950 is present in all the Border Collies, but only in 6/47 Boxers (Fig. 3b). Another four contigs were present in all Border Collies with penetrance >95% and showed variable amplification in Boxers with a penetrance ranging from 51% to 95% (Supplementary Table ST1). PCR confirmation indicates that these sequences represent novel sequence missing from the CGR and are not sequencing artifacts.

### Genome placement of de novo contigs

Mapping the raw reads to secondary assemblies using one-end anchor (OEA) reads in each breed provided genomic coordinates in the CGR for 90%, 99.57%, and 98.38% of contigs in Border Collie, Bearded Collie, and Entlebucher Sennenhund, respectively (Table 2). Considering only high quality unique Bowtie2 alignments (> = 30 mapping quality in both genome and assembly) resulted in genomic coordinates for 158,271 contigs (81.4%) in Border Collie, 6,055 (92.5%) contigs in Bearded Collie, and 13,119 (90.2%) contigs in Entlebucher Sennenhund. Among the high-quality mappings within the Border Collie samples, 138,361 (87.4%) contigs have single mapping loci, 16,947 (10.7%) contigs produced two genomic loci and 2963 (1.8%) contigs yielded more than two genomic loci. In Bearded Collie, 1,961 (32.3%) contigs mapped to single genomic loci, 1,661 (27.4%) mapped to two genomic loci, and 2,433 (40.18%) contigs mapped to multiple loci. In Entlebucher Sennenhund 7,399 (56.4%) contigs mapped to single loci, 3,008 (22.9%) contigs to two genomic loci, and 2,712 (20.7%) contigs to multiple loci (Supplementary Figs S3–5, Supplemental Datas 1 and 2). Among the 147,721 high-quality mappings with single genomic coordinates, 126,555 matched predictions with BLAT alignment and ranged throughout the genome and across functional gene regions in all three breeds.

**Table 2:** Total count of contigs with mapped genomic coordinates using paired-end relationships in all three breeds.

Breed	Total Contigs	Predicted contigs	High Quality Predictions	Single loci	Two loci	More than two loci
Border Collie	216240	194621	158271	138361	16947	2963
Bearded Collie	6569	6541	6055	1961	1661	2433
Entlebucher Sennenhund	14779	14540	13119	7399	3008	2712

### Sequences in CGR Gaps and BAC clones

Gap locations in the current CGR were retrieved using the UCSC Genome Browser mapping and sequencing track tool, which represent ~13.6 Mb of assembly gaps with 4.2 Mb (11,800 regions) of genic gap across 5,440 Ensembl, 4,970 RefSeq, and 7,129 canFam3.1+ genes (Table 3). A total of 126,555 contigs with predicted loci in agreement across OEA and BLAT alignment in the three breeds were examined for the presence or absence of gaps in the CGR. We found 11,555 gap contigs (contigs mapping to the gap regions in CGR) across the three breeds, filling 4.6 Mb of missing CGR sequence and, closing 12,503 known gaps. Within the gap contigs, 67% (7,711/11,555) overlap with exons, splice sites, UTR5/UTR3, introns or ncRNAs in 3,757 (11.5%) Ensembl, 3,405 (16.4%) RefSeq and 4,970 (12.2%) canFam3.1+ genes covering a total of 2.4 Mb corresponding to 7,444 unique genic gap regions in the CGR (Supplementary Figs S6 and 7). This includes 80 RefSeq, 248 Ensembl, and 383 canFam3.1+ genes with gaps within the CDS regions. Cross-referencing gap contigs with NCBI unique concordant and discordant BAC clone inserts (CHORI-82 Canine BAC Library CanFam3.1, DNA from CGR) revealed that 11,504 (99.5%) of the gap-contigs also spanned the BAC clone placements leaving 97 novel contigs spanning 88 gaps in the CGR without BAC clone inserts (Table 3, Supplemental Data 3**)** where few gaps are spanned by overlapping contigs.

**Table 3:** Summary of genes in CGR gaps spanned by BAC clone-inserts and assembled contigs.

	Ensembl	RefSeq	canFam3.1+
Gaps in CGR (11800)	9261	8061	8508
BAC clone inserts (11681)	5434	4966	7255
Contigs in assembly (7,711)	3757	3405	4970
Novel contigs in assembly (53)	25	21	32

Gaps overlapping an exon, splice site, UTR5/UTR3, intron, or ncRNA are classified as genic gaps.

The 97 novel gap contigs were examined for potential functional elements and the resulted annotations are summarized in Supplementary Table ST2. We found 53 novel contigs that close 47 known genic gaps within 25 Ensembl, 21 RefSeq, and 32 canFam3.1+ partial genes in the CGR. Contig bc_c101202 spanned the reported gap encompassing part of intron 5 through the beginning of exon 6 in a glutamate ionotropic receptor protein (*GRIN3B*). *GRIN3B* is only partially annotated in the RefSeq reference assembly and the contig has stronger similarity to human (93%) and mouse (89%) *GRIN3B* proteins than dog. Likewise, contigs bc_c91718, bc_c114599, and bc_c244765 spanned the gaps in the exons of eukaryotic translation elongation factor 1 alpha 2 (*EEF1A2*), zinc finger CCCH-type and G-patch domain containing (*ZGPAT*) and 5′, 3′-nuleotidase, cytosolic (*NT5C*) respectively. The remaining contigs contain introns, UTRs, or ncRNA from RNA-seq assembled transcripts in either ensembl, RefSeq, or canFam3.1+ annotations missing from the reference. Beyond these, 90% (115,000) of novel contigs mapped outside CGR gaps and could represent unidentified structural polymorphisms or breed-specific variation not found in the reference Boxer. These would most likely be novel sequence insertions and by definition they could be considered structural variants because they are not present in the reference genome. Contigs within and outside the known gaps can contain missing gene-rich sequences that may represent novel disease alleles or genes pertaining to intra- and interbreed specific traits and diseases^8^.

### Novel Allele Identification

**Figure 4:**
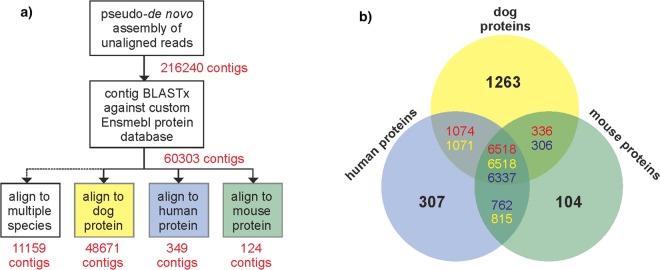
Annotation of Border Collie *de novo* contigs. (**a**) Flow chart of contig annotation. Number of contigs is indicated in red, contigs preferentially aligning to dog, human, or mouse protein Ensembl databases are indicated by colored boxes. Note: Some contigs aligned with equal weight to two databases. (**b**) Venn diagram of the number of protein coding genes to which novel contigs aligned. Numbers in the overlapping regions indicate the number of BLASTx hits aligning to dog (red), human (yellow), and mouse (blue).

We queried the 216,240 Border Collie *de novo* contigs against a database of all Ensembl annotated proteins in dogs, humans, and mice using BLASTx. 155,937 contigs had weak or non-existing alignments to any of the protein sequences in the database. Of the 60,303 aligned contigs, 48,671 aligned to only dog proteins, 349 contigs aligned to only human proteins, 124 contigs aligned to only mouse proteins, and 11,159 aligned to multiple species (Fig. 4a; Supplementary Table ST3). Genes represented within species-specific contigs include 1,263 dog, 307 human, and 104 mouse genes (Fig. 4b). Contigs with duplicate species hits are represented by 21,523 unique genes. Similar results were found in Bearded Collie and Entlebucher Sennenhund (Supplementary Figs S8 and 9). Contigs with BLASTx hits to dog proteins are candidates for novel alleles, while hits to human and mouse proteins are prospective novel alleles or splice variants of genes conserved across species and missing in the dog reference.

### Novel Contigs and Variants in Disease-associated Genes

**Figure 5:**
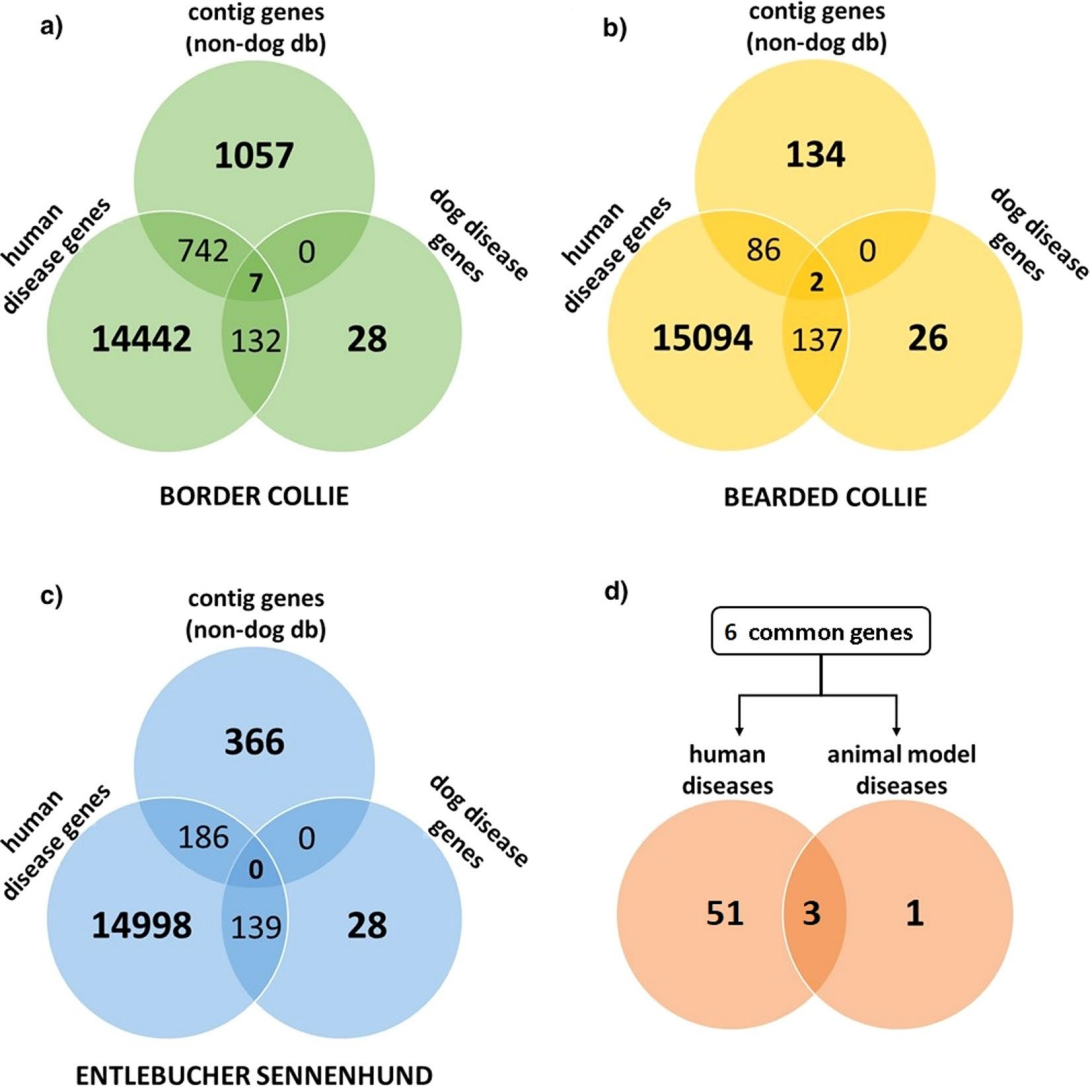
Known disease genes overlapping novel contigs. Overlap of non-dog genes aligning to contigs, genes associated with hereditary dog diseases (from OMIA), and genes associated with Mendelian human diseases (OMIM) in (**a**) Border Collies (**b**) Bearded Collies and (**c**) Entlebucher Sennenhunds. (**d**) There are 6 total genes represented in Border and Bearded Collie contigs that associate with diseases in humans and animal models. These genes are implicated in 54 human and 4 animal model disease phenotypes.

Novel contigs with translated sequence similarity closest to humans and mice are intriguing because these systems have been better studied for genomic variation associated with diseases. This disease association may be recapitulated in other breeds, but is not currently represented in the CGR. In total, we found 1,748 contigs among the three breeds that had stronger protein sequence similarity to human and mouse than dog protein sequence. D*e novo* contigs in Border Collie included 1806 unique non-dog gene hits of which 742 genes have been associated with human Mendelian diseases and 6 genes with both human and dog hereditary diseases with a disease-gene network database, disGeNET. (Fig. 5a, Table 4). Bearded Collie *de novo* contigs included 224 unique non-dog gene hits and 86 of those genes have been associated with human Mendelian diseases and 2 genes with both human Mendelian and dog hereditary diseases (Fig. 5b). Entlebucher Sennenhund *de novo* contigs included 336 unique non-dog gene hits with 186 of those genes associating with human Mendelian diseases and no genes associating with dog hereditary diseases (Fig. 5c). The six genes in Border Collies and two redundant genes in Bearded Collies are associated with 54 human and 4 animal model diseases (Fig. 5d). Table 4 summarizes examples of various types of novel content found in six genes that have been previously associated to known phenotypes in other breeds as identified through OMIA database.

**Table 4:** Examples of novel variants in six genes found in the Border and Bearded Collie contigs.

Contig ID	Gene ID	Contig Seq	Accession ID	Dog Disease	Affected Breeds	Human Disease
bc_87005	COL9A3	partial CDS in last exon due to gap in CGR	NP_001184100.1	Oculoskeletal dysplasia 1	Samoyed, Labrador Retriever	Stickler Syndrome, Type 1; Marshall Syndrome
bc_134555	DLX6	putative splice variant	NP_005213.3	Cleft palate 1	Nova Scotia Duck Tolling Retriever	Pierre Robin Syndrome
bc_156488	FAM161A	putative splice variant	XP_005626198.1	Progressive retinal atrophy, type 3	Tibetan Spaniel, Tibetan Terrier	Retinitis pigmentosa 28
bc_162349	RASGRP1	putative splice variant	AAX76907.1	Thrombopathia	Eskimo Spitz, Bassett Hound, Landseer Newfoundland	Storage pool platelet disease
bc_85943	RD3	putative splice variant	NP_898882.1	Rod-cone dysplasia 2	Rough Collie, Smooth Collie	Leber congenital amaurosis 12
bc_110123 & bc_92297	SCARF2	partial CDS in exons 4, 5, 12 due to gap in CGR	AAH00584.2	van den Ende-Gupta syndrome	Wirehaired Fox Terrier	van den Ende-Gupta syndrome

Table includes a description of the novel content within the contig, associated dog or human disease, and the affected breeds.

### Ab initio gene prediction in unknown contigs

AUGUSTUS, an *ab initio* gene finder was used to predict gene models in 49,455 (22.8%) Border collie *de novo* contigs without BLAST alignment to any of the known species in NCBI nt database (Fig. 2). AUGUSTUS predicted 301 gene models in 265 (0.53%) of the unaligned contigs. BLASTp of protein sequences from the 301 predicted gene models against NCBI nr database yielded hits to 32 proteins among 25 known species. Predictions with strong homology (> = 70% identity and > = 50 alignment score) were retained for further analysis (Supplemental Table ST4). Of note, we found contig bc_c77942 to have 100% homology to ceramide synthase 1 isoform X1 (XP_019271960.1) in *Panthera pardus* and bc_c192816 to have 97.5% homology to ceramide synthase 1 isoform X1 in *Sus scrofa*. Contig bc_c173637 was found to have 100% identity with beta-galactosidase precursor protein (NP_001032730.1) in *Canis lupus familiaris* and mutations in this gene are used as a canine model for late infantile human G-gangliosidosis^9^. *Ab initio* gene prediction in Bearded Collie and Entlebucher Sennenhund breeds did not yield any *de novo* contigs using the same criteria as performed with Border Collie.

## Discussion

The aim of this study was to discover and map potential sequences missing from the reference assembly and uncover novel genes or possible splice variants utilizing unmapped sequence reads. Despite the advancements in alignment algorithms, a substantial number of reads remain unmapped due to misassembly, absence of sequences in the reference assembly (alternate loci), assembly gaps and/or the limited number of individuals used to create the reference genomes. Moreover, recent studies in other species show that exploring unmapped reads often uncovers relevant biological information^10–13^. Consequently, we pooled reads from 41 dogs across three breeds and mined for novel genomic content using a pseudo-*de novo* assembly pipeline—an approach previously validated in humans and zebrafish—of non-mapping, high quality sequence reads which are usually discarded^10,11^.

We found 256,957 contigs with an N50 of 1,374 in Border Collies, 7,476 contigs with an N50 of 3,174 in Bearded Collies, and 15,455 contigs with an N50 of 2,156 in Entlebucher Sennenhunds. Novel contigs from all three breeds have a GC content similar to CanFam3.1 and high levels of complexity. Approximately 50% of the novel contigs are most similar to dog nucleotide sequence, 25% have closer nucleotide similarity to other species, and 25% have no similarity to any known nucleotide sequence. Annotation of the assembled contigs revealed that contigs spanned from 400 bp to 32 Kb and 13% of the total contigs overlapped the exonic sequence in the reference (Supplementary Figs S1 3–7). Interestingly, 10% of total contigs overlapped with gap regions in the CRG providing partially missing sequences for 11.5% of Ensembl, 16.4% of RefSeq, and 12.2% of canFam3.1+ genes across exons, splice sites, introns, UTR, and ncRNA regions in the CRG. Additionally, 91% (7,032) of gap contigs spanning genic gap regions overlap with gene models annotated using transcriptome data from 20 canine samples providing supporting evidence from RNA-seq data^14^, which should be incorporated into the CGR.

Novel sequences in disease-related genes not covered by BAC clones were identified to result from gaps preventing complete CGR alignment. Within the novel contigs, genes identified by BLASTn were conserved among mammals including human, mouse, rat, cow, chicken and zebrafish. One example, contig bc_c101202, contains glutamate ionotropic receptor NMDA type subunit 3B (*GRIN3B*) overlapping a gap (chr20:57705185-57705522) spanning the CDS region in the CGR. Protein sequence alignment of the contig showed similarity to human glutamate receptor ionotropic, NMDA 3B precursor protein (NP_619635.1) suggesting the presence of a gene isoform missing from the CGR. NMDA receptor dysfunction has been linked to neuropsychiatric disorders and a missense mutation in *GRIN3B* is implicated as an important risk factor for schizophrenia in humans^15^. Additional novel exonic non-BAC sequences within human disease related genes include: *EEF1A2*, which is associated with dilated cardiomyopathy^16^, intellectual disability, autistic behaviour and epilepsy^17^ and *ZGPAT*- epigenetic target for carcinogenesis^18^. Moreover, the novel gap contigs also include sequences overlapping splice sites, UTRs, introns, and ncRNA in 25 Ensembl, 21 RefSeq, and 32 canFam3.1+ genes. These targets remain unexplored and do not have BAC constructs. The inclusion of these sequences in the CGR will play a vital role in detecting genomic variations within the missing regions of genes that could carry putative disease alleles pertaining to simple and complex phenotypes. As an illustration, 88 genes that span CGR gaps are associated with obsessive compulsive disorder and autism spectrum disorder in humans, mice, and dogs based on 608 targeted genes^19^ indicating a pool of existing sequence variation that has yet to be explored in the dog model that may associate with those disease variations.

This analysis revealed 1,748 contigs spanning 2,366 genes, which represent variant alleles that are more similar to known human or mouse proteins than dog proteins and that are not annotated in the CGR. Using DisGeNET, a dynamic exploratory tool, we explored whether the novel contigs identified in this study may be associated with known genetic diseases^20^. Within the identified variant alleles, we found 1013 genes that are associated with human Mendelian diseases and 6 associated with both human Mendelian diseases and hereditary diseases in dogs. The six genes (*SCARF2*, *RD3*, *COL9A3*, *FAM161A*, *RASGRP1 and DLX6*) in common between novel contigs, human Mendelian diseases, and hereditary diseases in dogs are significant in known disease phenotypes in humans and animal models. To assess possible missing sequence in the CGR within these genes, we aligned the unmapped contigs to the Genbank dog assembly and probed protein sequence similarity to dog, human, and mouse genomes. The analysis uncovered that the raw reads did not map to the six genes either due to gaps, short insertions or deletions (indels) in the CRG, or previously unidentified splice variants.

Mutations in *SCARF2* gene are associated with unknown developmental syndrome in Wire Fox Terriers and established as a canine model for van den Ende-Gupta syndrome^21^. However, *SCARF2* is an incomplete gene with two gaps in the reference genome producing unmapped reads. Contig bc_110123 aligned to the reference sequence with one mismatch and spanned the gap (chr26:30233098-30233668) immediately downstream of *SCARF2* exon 12 (Supplemental Fig. S10). The sequence in the gap codes for 108 amino acids absent in the CRG and extends the current exon 12 to 296 amino acids prior to the stop codon. Canine exon 12 is found to be similar to human exon 11 of *SCARF2* transcript variant X1 which codes for 301 amino acids (AAH00584.2). Another contig, bc_92297, aligned to the reference sequence spanning another gap (chr26:30238633-302238823) between *SCARF2* exons 4 and 5 that codes for 41 amino acids missing from the CRG (Supplemental Fig. S11). The current canine exons 4 and 5 code for 131 amino acids and the contig extends the sequence to 172 amino acids. The gap currently breaks the sequence into canine exons 4 and 5, which otherwise is similar to exon 4 of human *SCARF2* transcript variant X1 with 172 amino acids.

A 1-base insertional mutation in exon 1 of *COL9A3* is reported to cause oculoskeletal dysplasia, which segregates as an autosomal recessive disorder in Labrador retriever and Samoyed dogs^22^. The current genome assembly (CanFam3.1) contains a gap (chr24:46667933-46668592, 659 bp) in this gene region with a 3 bp coding sequence ending immediately after the gap leading to the hypothesis that the genome assembly doesn’t correctly encode the complete CDS of *COL9A3*. Contig bc_87005 aligned to the CGR starting from chr24:46668600-46668878 with 96% identity and 666 unaligned base pairs at the 5′ end of the alignment start position (Supplementary Fig. S12). These 666 unaligned nucleotides in the gap region encode 55 amino acids and correspond to the ultimate CDS region of the RefSeq transcript NM_001197171.1 supported by dog mRNA GU075882.1 cloned by RT-PCR^22^. This validates our assembly and the accuracy of contig genome placement.

In the study by^23^, three splice variants of RD3 canine transcripts were detected with a total of 6 exons. Canine *RD3* splice variant three is composed of four exons of which canine exons 1 and 2 are non-coding and canine exons 3 and 4 correspond to coding exons 2 and 3 of human and mouse transcripts. The study found that canine exon 4 is missing from the canine assembly and mutations in exon 4 of *RD3* splice variant three are associated with rod-cone dysplasia type 2 in collie dogs. In our study, contig bc_85943 from the Border Collie assembly aligned to the transcript XM_022410347.1 of canine RD3 gene (chr7:9875357-9875470) with 4 mismatches and 1 gap and 1498 unaligned base pairs (Supplementary Fig. S13). Protein translation of the 1498 unaligned base pairs revealed the presence of partial exon 4 of *RD3* splice variant three as reported by Kukekova and colleagues^23^ and missing from the CGR. However, we found only partial sequence of exon 4, with our sequence ending at the hexamer repeat previously reported.

The *FAM161A* gene has been implicated in retinal degeneration in humans and progressive retinal atrophy in dogs. A study by^24^ showed that *FAM161A* is differentially expressed throughout the development of normal retina. We found a 447 bp insertion in the CGR after *FAM161A* exon 6 which leads to a stop codon after 51 amino acids. Contig bc_156488 (1,451 bp) aligned to the reference in two segments (chr10:61816040-61815425 and chr10:61814978-61814401 due to the insertion) which encoded for 188 amino acids before the stop codon of which 82 amino acids are similar to protein *FAM161A* isoform X3 (XP_005626198.1) of the mRNA transcript XM_005626141.3 (Supplementary Notes). This could imply the possible presence of breed-specific splice variant in *FAM161A* gene due to alternate splicing producing a different protein isoform. Contig bc_c162349 did not yield any BLASTn alignments to the CGR, but the translated sequence showed similarity to human Ras guanyl releasing protein I splice variant C (AAX76907.1) and Ras guanyl releasing protein I splice variant D (AAT47482.2), indicating the possible presence of a *RASGRP* ortholog missing from the CGR. Similarly, 376 bp of the contig bc_c134555 (1,186 bp) aligned to huntingtin isoform X2 in canine chromosome 3 while translated sequence showed similarity to human homeobox protein *DLX-6* (NP_005213.3) which implies the presence of putative homolog.

Altogether, we report an additional 2,360 genes representing variant alleles with higher similarity to human and mouse proteins missing from the reference and partially missing sequences for 11.5% of Ensembl, 16.4% of RefSeq, and 12.2% canFam3.1+ genes in CGR gaps. The inclusion of the missing genes sequences into the CGR will facilitate identification of putative disease mutations in diverse breeds and phenotypes. Further elucidation of breed-specific genetic diversity will improve screening, diagnosis, and treatment of genetic diseases in dogs. Moreover, it will enrich the application of the domestic dog as a biomedical large animal model for genetic diseases^25^. Our study suggests that sequencing 5–10 dogs per breed is adequate to identify common breed-specific contigs. The gain between 7–8 individuals (Bearded Collies & Entlebucher) to 26 individuals (Border Collies) only resulted in a ~36% increase of novel contig kb size. As efforts continue to refine the CGR, we recommend that the community should focus on the analysis of different breeds, but also acknowledge that rare variants can only be found by sequencing more individuals within a breed. Use of pseudo-*de novo* assembly of unmapped reads recovered sequence missing from the reference individual, Tasha, and serves as a baseline for improvement of canine reference assembly. These findings demonstrate that the current CGR lacks intra- and inter-breed-specific sequence variation and suggest the need for improvement of consensus CGR for the research community to avoid missing useful biological information. Much future work is warranted in discerning the allelic composition of all variants across dog species, especially for cryptic phenotypes or deleterious alleles present at low frequency within populations. Cost-efficient short read sequencing is now a common approach with an exponentially increasing number of full genome sequences available across a diverse range of species. The availability of these sequences makes pseudo-*de novo* analyses of unmapped reads a feasible alternative to other, more costly long-read platforms (e.g., PacBio). While long read technologies may facilitate a more complete capture of individuals CNV, insertions, deletions, and other structural variants and improve reference genome assembly, the availability of currently published genome sequences and lower cost of generating new short read genome sequences will continue to make this methodology a valuable contributor of reference genome analysis as demonstrated here with the CGR.

## Methods

### Sample Collection and Sequencing

EDTA-blood samples from 22 Border Collies were collected and stored at −20 °C prior to genomic DNA isolation using a semi-automated Chemagen extraction robot (PerkinElmer Chemagen Technologie GmbH). DNA concentration was determined with a NanoDrop ND-1000 UV/Vis Spectrophotometer or Qubit 3.0 Fluorometer (Thermo Fisher Scientific Inc.). Whole genome sequencing was performed by the Science for Life Laboratory (Stockholm, Sweden) on an Illumina HiSeq2000 producing 100 bp paired-end reads with a 290 bp insert size with >20Xcoverage in 22 Border Collie samples. Public sequence data of 4 Border Collies, 7 Bearded Collies and 8 Entlebucher Sennenhunds (12–20X) were downloaded from the studies^26,27^ (Supplementary Table ST5). The three breeds used in this study were selected due to the availability of high quality sequencing data (>5 individuals per breed) and because the three breeds represent a distinct group of breeds (herding breeds) with genomes expected to be highly diverging from the boxer reference (working breed). Sample collection was approved by the Animal Ethics Committee of State Provincial Office of Southern Finland, Finland (ESAVI/7482/04.10.07/2015). All experiments were performed in accordance with relevant guidelines and regulations.

### Pre-processing

Illumina adapters were removed using the ILLUMACLIP tool in trimmomatic^28^ using the following parameters: seed mismatches = 2, palindrome clip threshold = 30, and simple clip threshold = 10. Raw reads were trimmed for phred >20 using a sliding window of 4 bases with low quality bases (quality score >3) removed from the leading and trailing ends. All reads less than 50 bp were discarded. Quality assessment of reads was performed pre-and post-processing using FastQC (http://www.bioinformatics.babraham.ac.uk/projects/fastqc/ by S. Andrews). Trimmed, paired reads were then split and aligned to the canine reference genome CanFam3.1^29^ using bowtie2^30^. Reads that did not align concordantly or did not align at all were extracted as unaligned reads and use for downstream pseudo-*de novo* assembly.

### Pseudo-de novo Assembly Pipeline

Trimmed reads that failed to map to the reference genome were *de novo* assembled into contigs for each individual using the default parameters *genome*, *denovo*, *accurate* using MIRA 4.0^31^, a multi-pass DNA sequence data assembler that performs well on difficult assembly tasks such as repetitive and orphan reads^10,11^. BLASTn^32^ on 10,000 random contigs revealed possible sample contamination with two species of roundworm (*Onchocerca ochengi* and *Parascaris equorum*) in Border Collies and two strains of bacteria (*Pantoea agglomerans* and *Serratia plymuthica*) in Bearded Collies and Entlebucher Sennenhunds. As a result, contaminated reads were removed and cleaned unaligned reads were reassembled. A population-level *de novo* assembly was then performed in MIRA 4.0 using all assembled contigs complied from each individual by breed. Primary contigs that were identified as singlets during the secondary assembly were binned as debris and excluded. Secondary assembly contigs were evaluated for GC content, complexity, and duplication using PRINSEQ^33^. Non-BAC end Sanger sequencing reads for the original Boxer were retrieved from NCBI Trace Archive (CENTER_PROJECT = G630) and aligned to the reference genome using Bowtie2. The unmapped Boxer reads were then aligned to the secondary assemblies of each breed to identify the fraction of the assembled contigs representing errors/gaps in the assembly versus breed-specific variation.

### Genomic Loci Mapping

To determine the genomic loci coordinates of the *de novo* contigs in the reference genome, we utilized the genome mapping method implemented in Faber-Hammond and Brown 2016a. Briefly, raw reads from each dog within a breed were realigned to the secondary assembly of novel contigs and CGR using Bowtie2 to generate the SAM outputs for both the genome and assembly. One-end anchor (OEA) reads—sequences where only a single mate is aligned—were extracted from the genome and assembly SAM outputs. Genomic coordinates for the novel contigs were then predicted by cross-referencing the OEA mapping coordinates. Unique read mappings with scores of > = 30 both in genome and assembly are used to filter for high quality mapping loci. 5′ and 3′ coordinates of each contig were used to classify high quality mapping loci into single mapping loci and multi-mapping loci. Contigs with high quality single mapping loci were further validated by performing BLAT alignment of the contigs to the genbank reference genome to accurately map the genomic coordinates of the novel contigs in the CGR. The genomic location of the contigs were annotated using RefSeq, Ensembl and canFam3.1+ ^14^ gene models and further used to assess the contigs that encode sequence variation detected within each breed. Despite the overall high-quality of the CGR assembly (canFam3.1), it is known that ~1% of the euchromatic portion of the genome contain gaps due to allelic variation, assembly errors, simple sequence repeats, and high GC content which limits our understanding of canine genetic variation and evolution^29,34^. Gap locations in the current CGR were retrieved using the UCSC Genome Browser mapping and sequencing track tool to identify the missing sequences in the CGR and present in the assembly^35^.

### PCR Validation

PCR primers were designed using Primer3 program (primer3.sourceforge.net) to verify eight pseudo-*de novo* assembled contigs (Supplementary Table S1). Contigs were selected by filtering for those containing CNV across gene regions that lack simple repeats and microsatellites. DNA from 47 border collies and 47 boxers was used to confirm novel contig identification between the two species. PCR was performed on a Bio-Rad T100 Thermal Cycler (Bio-Rad Laboratories, USA) under the following conditions: denaturation step at 95 °C for 5 min, followed by 35 cycles of 95 °C for 30 s, annealing for 30 s at temperatures (58–61.8 °C) optimized for each primer pair, synthesis at 72 °C for 45 s and a final elongation at 72 °C for 10 min. Amplification products were run on a 1% agarose gel and scored for presence or absence in one or both breeds.

### De Novo Assembly Contig Annotation and Disease Association Analysis

BLASTx on assembled contigs was performed to identify potential orthologs to translated human (Homo_sapiens.GRCh38.pep.all.fa.gz), mouse (Mus_musculus.GRCm38.pep.all.fa.gz), and dog (Canis.familiaris.CanFam3.1.pep.all.fa.gz) protein-coding sequences retrieved from the Ensembl ftp database. Results were sorted and filtered base on a shared sequence identity >70% and an alignment score >50 to purge low-confidence hits. Contigs with stronger hits to human or mouse protein sequences as compared to dog were sorted and filtered on e-value and length. Unique non-dog BLASTx results were converted from Ensembl to Entrez annotation using bioDBnet^36^. We also curated a list of known hereditary diseases in dogs for comparison. To create this list, we generated a list of known diseases in dogs from the Online Mendelian Inheritance in Animals (OMIA) database (http://omia.angis.org.au) and translated OMIA IDs into Online Mendelian Inheritance in Man (OMIM) IDs. We assumed that overlap between unique non-dog BLASTx results and genes involved in hereditary diseases in dogs would likely be strong candidates for newly identified hereditary disease-relevant genes and alleles in dogs. To confirm this, we queried this list for known gene associations in human disease using the “CURATED” database or for known gene associations in animal models for disease using the “PREDICTED” database within the disGeNET R package^20^. Genes shared between human and animal model disease databases are top candidates for novel genes and alleles relevant for studying genetic diseases in dogs.

### Ab initio Gene Prediction

*De novo* contigs without BLAST alignment in each breed were inspected further for the presence of gene models using AUGUSTUS v.3.2.3^37^, a Hidden Markov Model-based *ab initio* gene predictor. The default Human training parameters provided with AUGUSTUS were used for predicting the genes in the *de novo* contigs, as recommended by the supporting documentation (and via personal correspondence between LAH and the primary author of AUGUSTUS).

### Data Availability

Raw whole genome sequence reads for Border Collie have been submitted to NCBI SRA and Novel contigs are archived at NCBI Assembly. Supplementary Data 1–3 are available at, https://drive.google.com/open?id=19y_cJlY8s3Awk2YFUjYgMXqIzSYCVq3C.

## Electronic supplementary material

Supplementary Tables 1,2,4,5

Supplementary Table 3

Supplementary Information
