# Author Correction: Assembly and Analysis of Unmapped Genome Sequence Reads Reveal Novel Sequence and Variation in Dogs

**DOI:** 10.1038/s41598-018-30169-3

**Published:** 2018-08-02

**Authors:** Lindsay A. Holden, Meharji Arumilli, Marjo K. Hytönen, Sruthi Hundi, Jarkko Salojärvi, Kim H. Brown, Hannes Lohi

**Affiliations:** 1 Department of Biology, Portland State University, Portland, Oregon, USA; 2 Research Programs Unit, Molecular Neurology, University of Helsinki, Helsinki, Finland; 3 Department of Veterinary Biosciences, University of Helsinki, Helsinki, Finland; 4 Folkhälsan Institute of Genetics, Helsinki, Finland; 5 Research Programme on Individuals and Populations, Faculty of Biological and Environmental Sciences, University of Helsinki, Helsinki, Finland; 6 School of Biological Sciences, Nanyang Technological University, Singapore, Singapore

Correction to: *Scientific Reports* 10.1038/s41598-018-29190-3, published online 18 July 2018

In this Article, the Data availability statement contains an incorrect link to the Supplementary Data 1-3. The correct Data availability statement is shown below:

“Novel contigs and Supplementary Data 1–3 are available at, https://pdxscholar.library.pdx.edu/bio_data/3/. Raw whole genome sequence reads for Border Collie dogs used in this study are available upon request.”

