# Supplementary material for: Assembly and Analysis of Unmapped Genome Sequence Reads Reveal Novel Sequence and Variation in Dogs
Source: Sci Rep. 2018 Jul 18;8:10862. doi: 10.1038/s41598-018-29190-3 (PMC6052005; doi:10.1038/s41598-018-29190-3)
Supplement: Supplementary file 3 — Supplementary Information [file 41598_2018_29190_MOESM3_ESM.pdf]

# Assembly and Analysis of Unmapped Genome Sequence Reads Reveal Novel Sequence and Variation in Dogs

Lindsay A. Holden[1+], Meharji Arumilli[2,3,4+], Sruthi Hundi[2,3,4], Marjo K. Hytönen[2,3,4], Jarkko Salojärvi[5], Kim H. Brown[1*], and Hannes Lohi[2,3,4*]

[1] Department of Biology, Portland State University, Portland, Oregon, USA

[2] Research Programs Unit, Molecular Neurology, University of Helsinki, Helsinki, Finland

[3] Department of Veterinary Biosciences, University of Helsinki, Helsinki, Finland

[4] Folkhälsan Institute of Genetics, Helsinki, Finland

[5] Research Programme on Individuals and Populations, Faculty of Biological and Environmental Sciences, University of Helsinki, Helsinki, Finland

+Denotes equal contribution of First Authors

[*]Denotes equal contribution of Senior Authors

Authors for Correspondence:

Hannes Lohi, Research Programs Unit, Molecular Neurology, University of Helsinki, Helsinki, Finland , hannes.lohi@helsinki.fi

Kim H Brown, Department of Biology, Portland State University, Portland, Oregon, USA, kibr2@pdx.edu

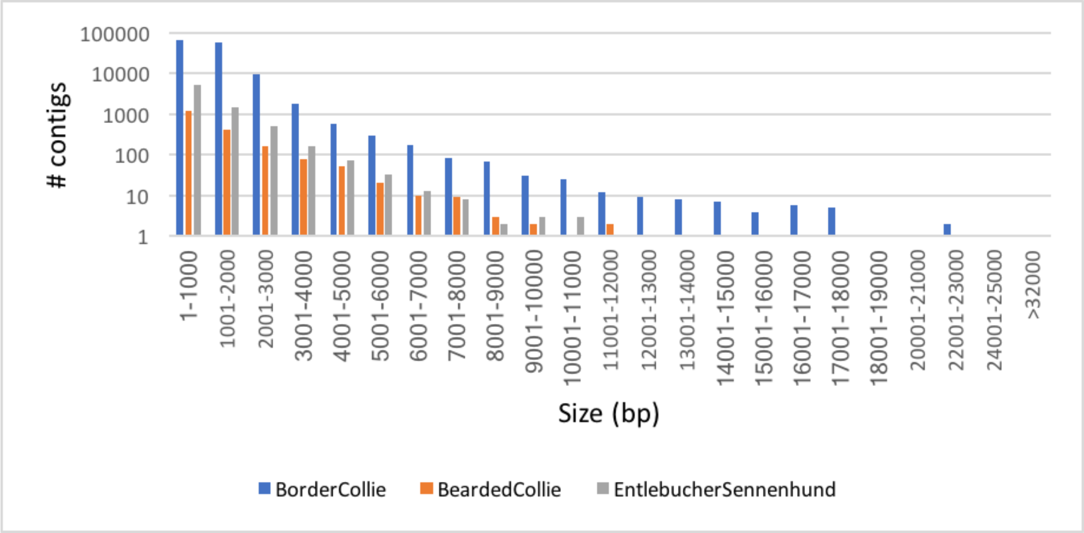


**Supplementary Fig. S1**. Size distribution of contigs across the three breeds

**a**

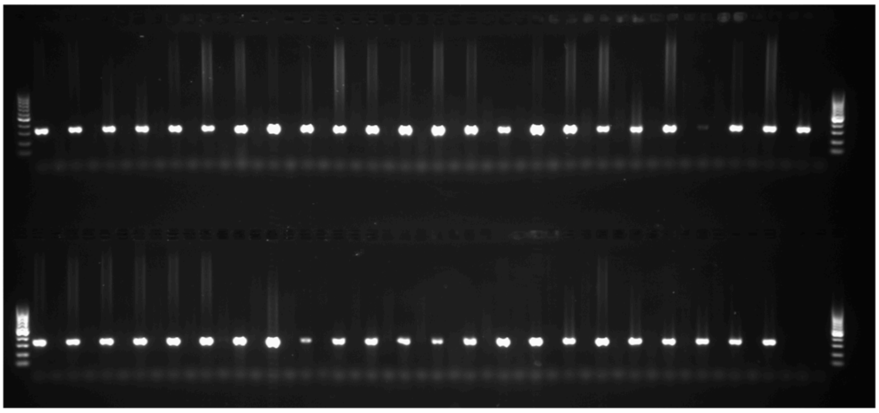

**b**

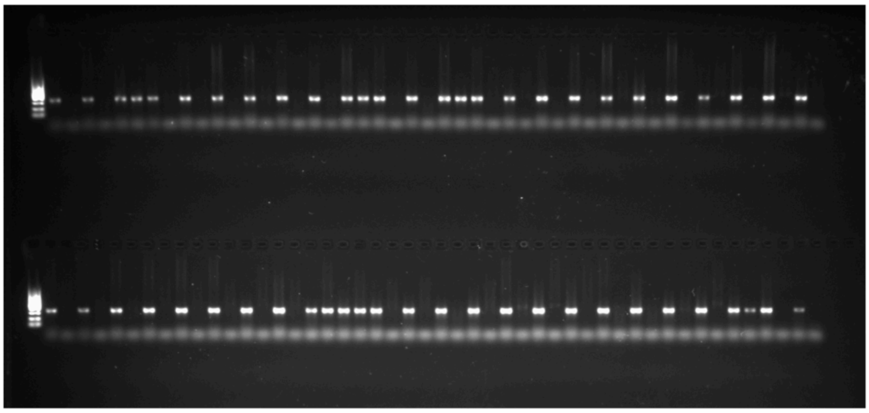

**Supplementary Fig. S2**. **Polymerase chain reaction (PCR) examples of the contigs.** a) Full-length gel image showing the amplification of the contig bc_c107501 in all Border Collies (BC) and no Boxers (B). b) Full-length gel image showing variable amplification of the contig bc_c222950 in Border Collies and Boxers.

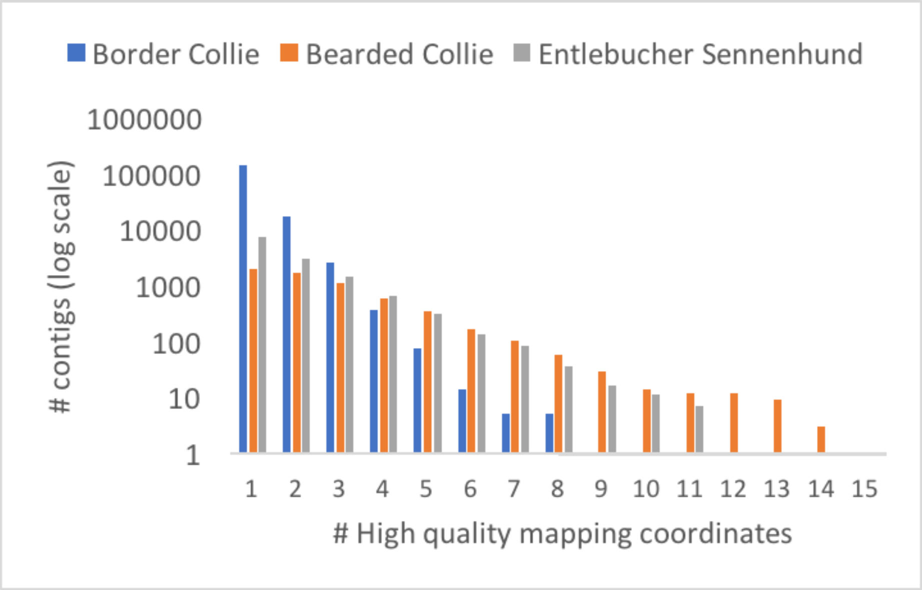


**Supplementary Fig. S3**. Predicted number of loci for contigs with Bowtie2 alignment scores ≥30 in both genome and assembly. Figure shows count of contigs mapping to one or more chromosomal loci.

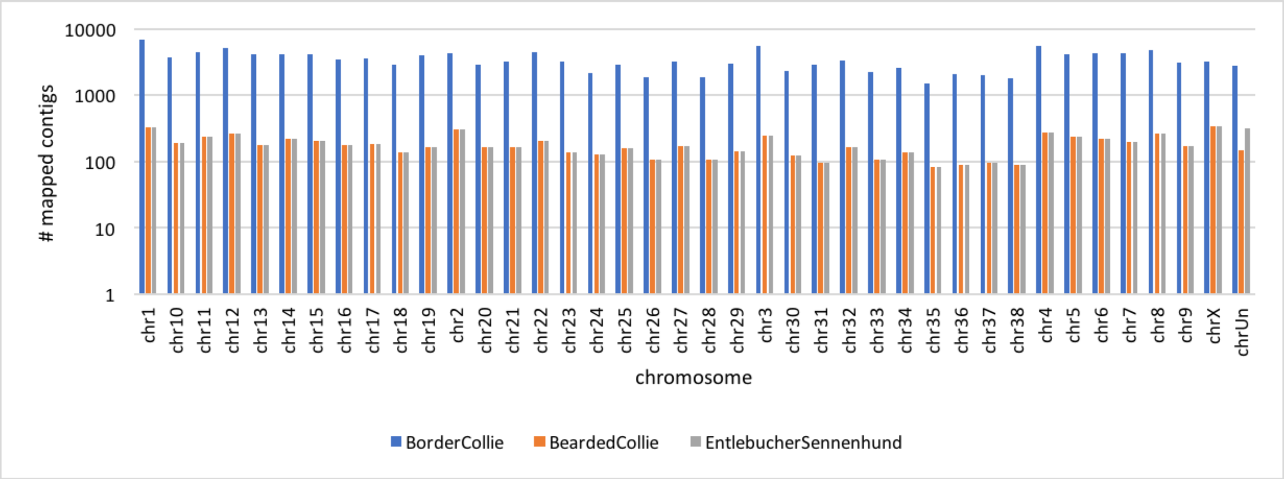


**Supplementary Fig. S4**. Distribution of the location of the contigs in canFam3.1 assembly based on the predicted loci across the three breeds.

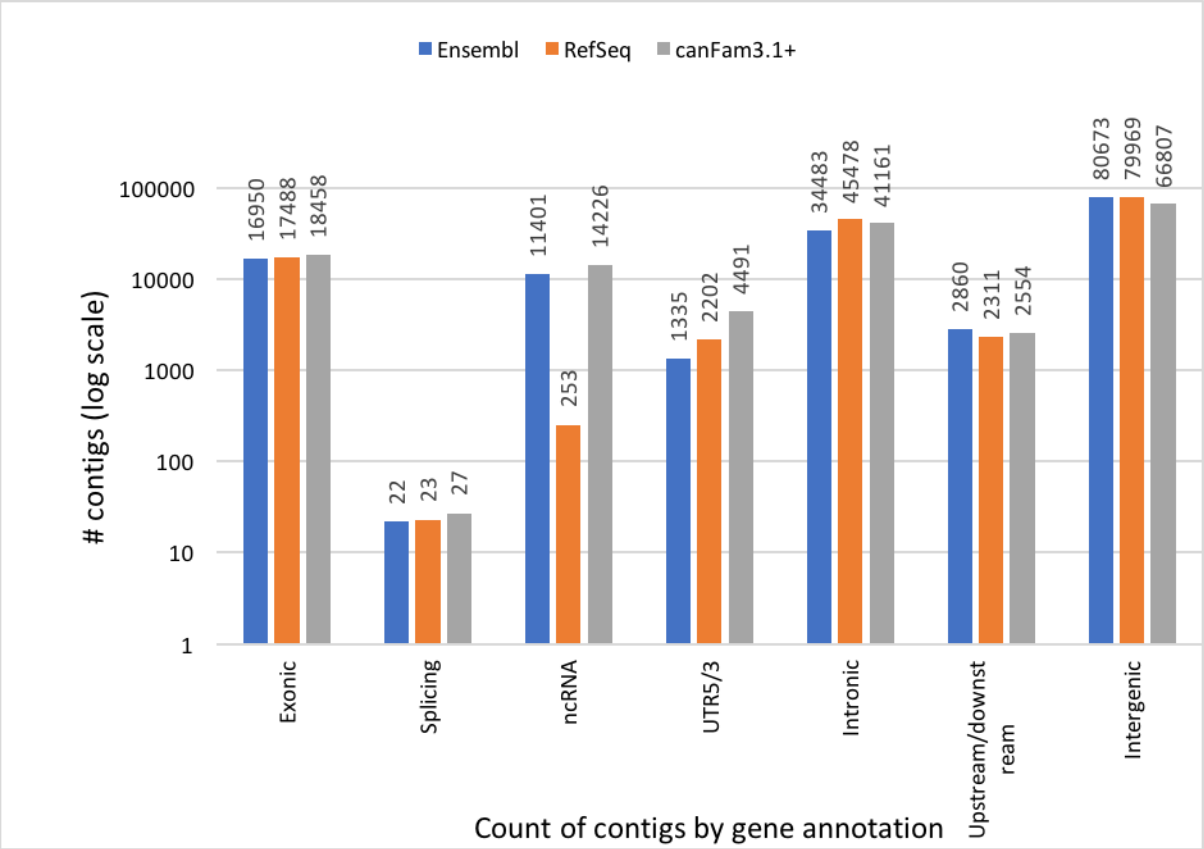


**Supplementary Fig. S5**. Histogram showing the count of the total contigs across functional regions in the genome based on Ensembl, RefSeq and canFam3.1+ annotations.

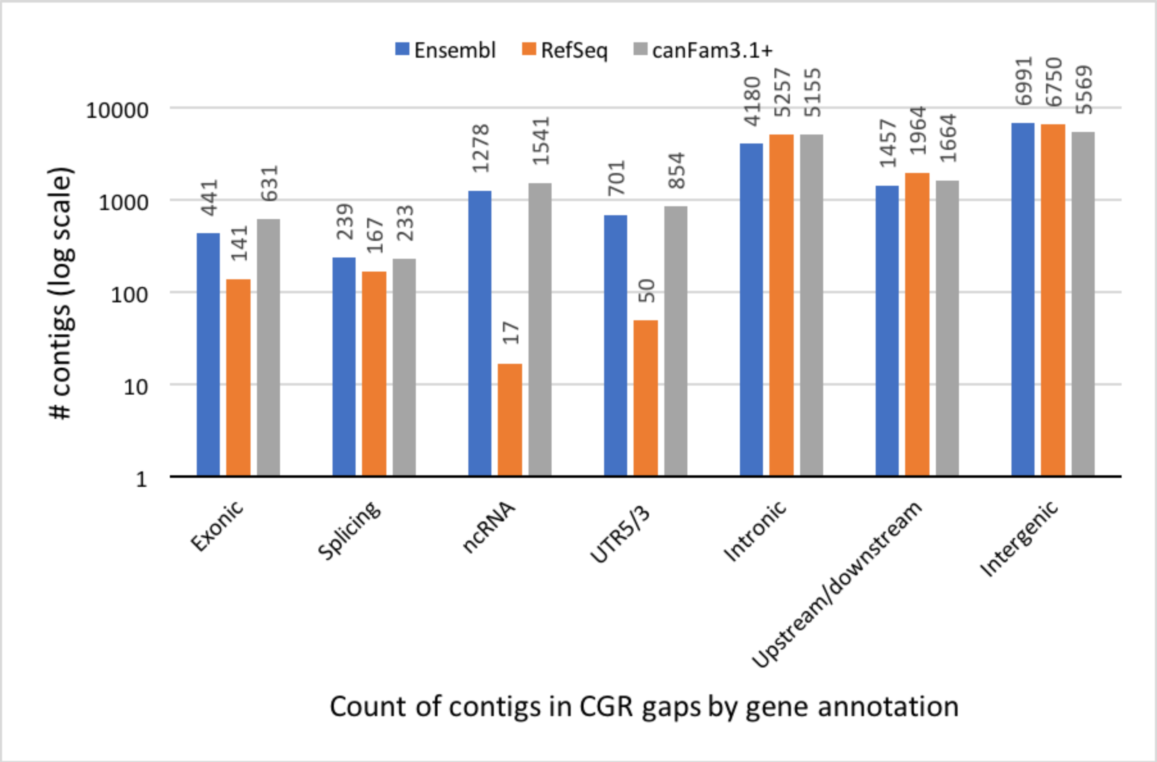


**Supplementary Fig. S6**. Histogram showing the count of the contigs embedded within CGR gaps across functional regions in the genome based on Ensembl, RefSeq and canFam3.1+ annotations.

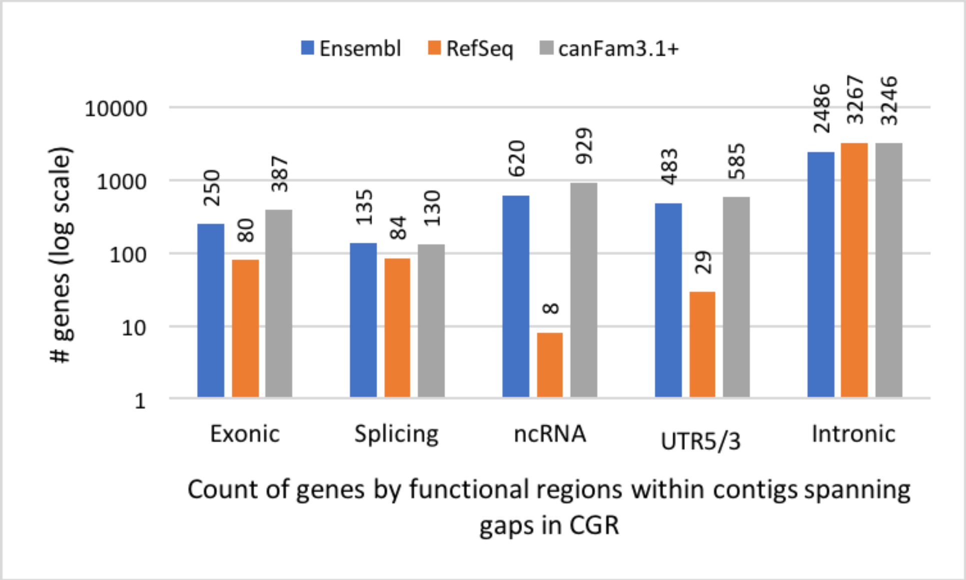


**Supplementary Fig. S7**. Histogram showing the count of the genes embedded within CGR gaps across functional regions in the genome based on Ensembl, RefSeq and canFam3.1+ annotations.

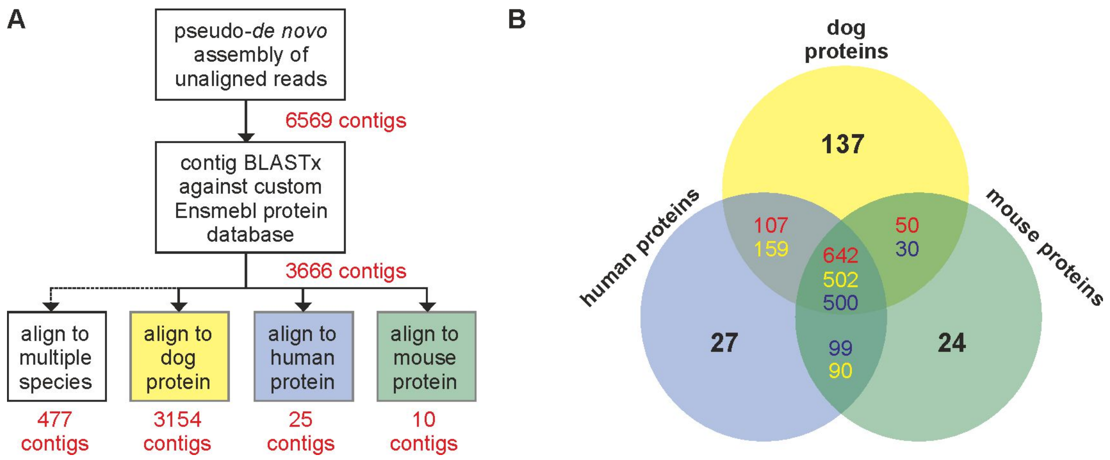


**Supplementary Fig. S8. Bearded collie Contig annotation.** a) Flow chart of contig annotation. Number of contigs is indicated in red, contigs preferentially aligning to dog, human, or mouse protein Ensembl databases are indicated by colored boxes. Note: Some contigs aligned with equal weight to two databases. b) Venn diagram of the number of protein coding genes to which novel contigs aligned. Numbers in the overlapping regions indicate the number of BLASTx hits aligning to dog (red), human (yellow), and mouse (blue).

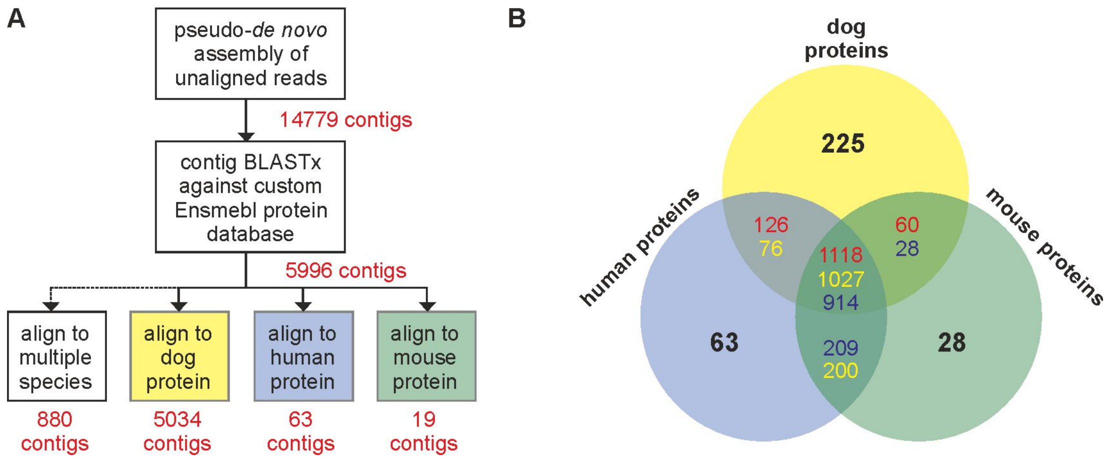


**Supplementary Fig. S9. Entlebucher Sennenhund contig annotation.** a) Flow chart of contig annotation. Number of contigs is indicated in red, contigs preferentially aligning to dog, human, or mouse protein Ensembl databases are indicated by colored boxes. Note: Some contigs aligned with equal weight to two databases. b) Venn diagram of the number of protein coding genes to which novel contigs aligned. Numbers in the overlapping regions indicate the number of BLASTx hits aligning to dog (red), human (yellow), and mouse (blue).

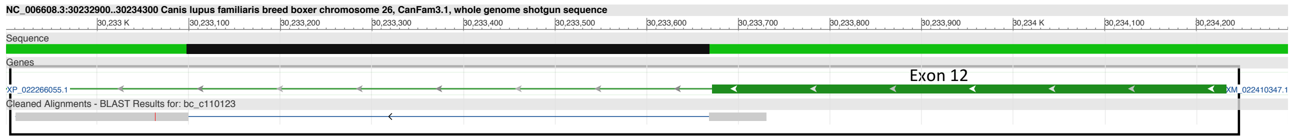


bc_c110123

```
5'
30234240
^gagaccgcagccgagagccgggacgcggagcccccacggcccctgccgaggcgccggcg
 E  T  A  A  E  S  R  D  A  E  P  P  T  A  P  A  E  A  P  A
Accctgcccgcgt{ccgcagaggaggcgacgcccctccccgcgtcctcggacagcgagcgg
 T  L  P  A   S  A  E  E  A  T  P  L  P  A  S  S  D  S  E  R
tcggcgtcgagcgtggacgggcccggcggggcgctgtacgcgcgcgtggcccggcgcgag
 S  A  S  S  V  D  G  P  G  G  A  L  Y  A  R  V  A  R  R  E
gcccggccggcccgggcccggggcgaggcgggggcctgtctctctcgccatcccccgag
 A  R  P  A  R  A  R  G  E  A  G  G  L  S  L  S  P  S  P  E
cgcaggaagccacctccacccgaccctgccaccaagcccaaggtgtcctggatccacggc
 R  R  K  P  P  P  P  D  P  A  T  K  P  K  V  S  W  I  H  G
aagcacggcgccgctgccgctgcccgcgcgccgtccccgccgctcccgggacccgaggcc
 K  H  G  A  A  A  A  A  R  A  P  S  P  P  L  P  G  P  E  A
gcgcccagccccagcaagaggaaacggacgcccagcgacacgtcggcgcggccggaggag
 A  P  S  P  S  K  R  K  R  T  P  S  D  T  S  A  R  P  E  E
cccggcagcccccgggcccgcgacccgacgccccggcccccggggctggcggaggaggcg
 P  G  S  P  R  A  R  D  P  T  P  R  P  P  G  L  A  E  E  A
Ccagccctggcctcccccctcgccgccccgggctcgggcgcggggccgcggccccggcctc
 P  A  L  A  S  P  S  P  P  R  A  R  A  R  G  R  G  P  G  L
tcggagcccacggacgcgggcggcccccgcgcagcgcgcccgaggccgcctccctgctg
 S  E  P  T  D  A  G  G  P  P  R  S  A  P  E  A  A  S  L  L
gcggcggagctgcgcgacaagactcgcagcctgggccgcgccgagggggctccgggcgcg
 A  A  E  L  R  D  K  T  R  S  L  G  R  A  E  G  A  P  G  A
cagggcccgcgggagaagccggcgccgccgcagaaggccaagcgctcggtgctgcccgcc
 Q  G  P  R  E  K  P  A  P  P  Q  K  A  K  R  S  V  L  P  A
tcgccggcgcgcgcgggccctgcgcccgaggccccggggcccgagaaggcggcggccggc
 S  P  A  R  A  G  P  A  P  E  A  P  G  P  E  K  A  A  A  G
gcgcccgcgcccgacacccccccggaagaagacccccatacagaagccgccgcgcaagaag
 A  P  A  P  D  T  P  R  K  K  T  P  I  Q  K  P  P  R  K  K
agccgggaggcggcgggcgagccgggcagggccggcgcccccaccctg}tagcgggcggcg
 S  R  E  A  A  G  E  P  G  R  A  G  A  P  T  L  *
gcccggcgcgcccgcaggttccccaggcttagcagcgctgcttgccaccccgcgtcccgc

gcccgcccgacgtgcccgcggcggcctcccggggcctcggcccggagccgcggggcggga

ggccgcgtatcgtatcgtcggctccggctcccacgggcgctcagcaggcccctccccgcc

ccttggccgagggctccgagggctccgagggctccgccgggcgggggtcccgagggccga

ggacccgagggttccgcctggagaccgcgtctcacggacccggagcgcgcccggcggggc

gccttctcattggttgaggccagacgcacttccgcctcggccgcctctcattggtcgggg

Cctggcgccctggccgggggcctctcctagacggtagcccagggctacttccggcagccg

ggctgctcccgctggcccgcccgggcgtctggtgg
                                   3'
                                   30232911
```

**Supplementary Fig. S10.** Upper panel showing the sequence alignment of contig bc_110123 to the reference sequence chr26:30232911-30234240 with one mismatch spanning the gap chr26:30233098-30233668 which is downstream of exon 12 in SCARF2 gene. Lower panel demonstrating the sequence alignment of contig

bc_110123 that codes for 108 amino acids missing in the reference. The contig extends the current exon 12 with 188 amino acids to a total 296 amino acids before the stop codon (*). The sequence in {} represent overlapping sequence with partial AAH00584.2 SCARF2 human protein. The underlined nucleotides represent the complete sequence in the contig and '^' represents the beginning of exon 12 in the reference. The green nucleotides represent the sequence in the reference, black nucleotides represent the sequence in the gap filled by contig.

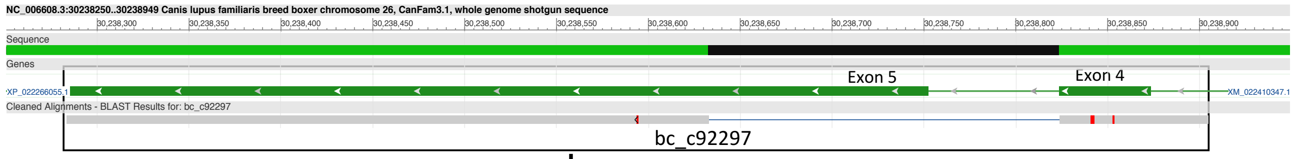


5' 30238904

```
ggcccgacaccctcctcgcccgcccctgca^gagtgcccgcgccagttctggggccccgac
 G  P  T  P  S  S  P  A  P  A   E  C  P  R  Q  F  W  G  P  D
tgcaaggagctgtgtatgtgccacccgcacgggcagtgcgaggacgtgacaggccagtgt
 C  K  E  L  C  M  C  H  P  H  G  Q  C  E  D  V  T  G  Q  C
acgtgtcacgcgcggcgctggggcgcacgctgcgagcatgcgtgccagtgccagcatggc
 T  C  H  A  R  R  W  G  A  R  C  E  H  A  C  Q  C  Q  H  G
gtgtgccacccgcggagcggcgcgtgtcgctgcgagcccggctggtggggcgcgcagtgc
 V  C  H  P  R  S  G  A  C  R  C  E  P  G  W  W  G  A  Q  C
gccagcgcgtgctactgcagcgccacgtcgcgctgcgacccacagacgggcgcgtgcctg
 A  S  A  C  Y  C  S  A  T  S  R  C  D  P  Q  T  G  A  C  L
tgccacgcaggctggtggggccgcagctgcaacaatcagtgcgcctgcaacacgtcgccg
 C  H  A  G  W  W  G  R  S  C  N  N  Q  C  A  C  N  T  S  P
tgcgagcagcagagcggccgctgccagtgtcgcgagcgcactttcggcgcgcgctgcgag
 C  E  Q  Q  S  G  R  C  Q  C  R  E  R  T  F  G  A  R  C  E
cgctactgccagtgctttcgcggccgctgccaccctgtggacggcacgtgcgcctgcgag
 R  Y  C  Q  C  F  R  G  R  C  H  P  V  D  G  T  C  A  C  E
ccgggctaccgcggcaagtactgccgggagccgtgccccgccggcttctacggcctgggc
 P  G  Y  R  G  K  Y  C  R  E  P  C  P  A  G  F  Y  G  L  G
tgccgccgccg
 C  R  R
```

3' 30238284

**Supplementary Fig. S11**. Upper panel showing the sequence alignment of contig bc_92297 to the reference sequence chr26:30238284-30238904 with 4 mismatches and spanning the gap chr26:30238633-30238823 which is downstream of exon 4 and upstream of exon 5 in SCARF2 gene. Lower panel: Sequence alignment of contig bc_c92297 demonstrating the gap sequence that codes for 41 amino acids between exon 4 and exon 5 of SCARF2 gene which is missing in the canine reference. The underlined nucleotides represent the complete sequence in the contig and '^' represents the beginning of exon 4 in reference. The green nucleotides represent the sequence in the reference and black nucleotides represent the sequence in the gap filled by contig.

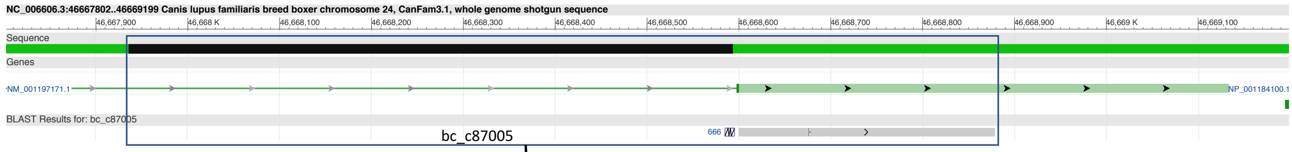


5’ 46668600

```
cgcgtacccgggatgctgtcaggagcagcgccccccccgccccgtccatccccccagccgc
acacacacgtcacctaggacagcgggggtcacgctcacacccccctcctcccctgcaggcc
ccgcccacgccccccagccgaccccatgcctccctgtgctcaggggacccgtccctgagg
tgggggcccccgggctccactgcagcacccccatccctgctcaccaagcagctctgcaca
cgtacccctcgcccccgaaagaccctgtcttcactcgagctgccatgctcggggcagacg
ccaggcctccacggcctccccacctcgtcctccgcgccgcctcctgccctcggcccttcc
agagggaggaagcagcccctaagcagcagggctgggggtggcgggctgaacggatggaag
tcaccaccgacgtcctcttctcccgtcaacactcaccttcccgctccgcctcca{ggacca
                                                      G  P
caaggcgtgcctggcgttagcaaagacggccgcgacggggccaacggcgagcccgggcct
 Q  G  V  P  G  V  S  K  D  G  R  D  G  A  N  G  E  P  G  P
ccaggcgatcctggcctccccggtgctgtgggtgctcaggggacacccggcatctgcgac
 P  G  D  P  G  L  P  G  A  V  G  A  Q  G  T  P  G  I  C  D
acctcggcctgccaaggagctgtgatgggaggcggcggggaaaagtcaggttctagaagc
 T  S  A  C  Q  G  A  V  M  G  G  G  G  E  K  S  G  S  R  S
ttc}taaaacgtaaccaaaggaactgaggtggtggtgccgatgccagcagggagctctgga
 F  -
gggacgccaggagtcggcggcactctgtgccgggaggggacagcatgccacccgcggcag
ccccgcggccctcctggcctgcccctcgttcctcggtggcagcagccggacacttgtcc
ctgagagcatccgtcatagacctgttgcccagtgtcgtcctccgtgcatctccacctgtg
catcagctctaacatcgtacctcgggcacgggtcccgtgtgcacgaccgaga
```

3’ 46668878

**Supplementary Fig. S12.** Upper panel showing the sequence alignment of 288 base pairs of the contig bc_87005 to chr24:46668600-46668878 with 666 unaligned base pairs at the 5’ end of the contig. Lower panel demonstrating the presence of ultimate CDS region of the transcript NM_001197171.1 in COL9A3 that codes for 55 amino acids before the stop codon. The underlined nucleotides represent the complete sequence of the contig while the light green nucleotides represent the non-coding sequence, the dark green nucleotides represent the coding sequence and the black nucleotides represent the sequence of the contig within the gap.

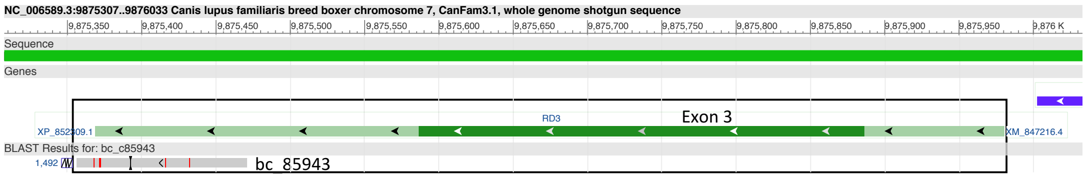


```
5'
9875357
tccttcctcccccttttcctccccccccaactcaaccgacagagcggcccaagagagacgcac

tcccgccctgcgtcagcagggccgaggggggccatggccctcatcccctggcttcgctgg
                                  M  A  L  I  P  W  L  R  W
aatgaagccccccgcggctgtcccccggaggccggctgagatggtgctggagacgctc
 N  E  A  P  P  R  L  S  P  R  R  P  A  E  M  V  L  E  T  L
atgatggagctggcggggcagatgcgggaggccgagaggcagcaatgggagcgcagtaac
 M  M  E  L  A  G  Q  M  R  E  A  E  R  Q  Q  W  E  R  S  N
gcgctcaggaaggtctgcaccggcgtggactacagctggctggccagcgcgccgcggccc
 A  L  R  K  V  C  T  G  V  D  Y  S  W  L  A  S  A  P  R  P
acctacgacctcagccctggcgagcggctgcagctagaggacgtctgtgccaagatccac
 T  Y  D  L  S  P  G  E  R  L  Q  L  E  D  V  C  A  K  I  H
ccatcctactgcgggcccgccatcctcaggtaacccccacccccctcccaggccaggcct
 P  S  Y  C  G  P  A  I  L  R  -
tgcaccgcaggctgcggcggtgtggctgcaggggcaggggcccgaggcccccaggacgcg

agccagggcccctggatgacccagggacc{tgtaaaaggagcggcaccgcaggctgcaggg

gtgtggcggcaggggcctgaggcacccaggatggcagccagggcccctggatgacccag

agatctgtaaaaggaacagggcagaagcaagactctgcaagagcccatagaacaccaggc

cagagccggggttcagagggaggcagcaggcaggaggcacatgacttttgatgggagagg

accccaggcctttgagctttctgttctccctggattatcattgccccggattttaacaca

gcgggctctttctccttcgtcggttcatcttccatattcccttcttagaaaggctttcca

gatagtcttcccagagttcagtcttcagtatcctagttcacactagcattcgtattagtt

tgcctcaagggactatgcgtgcatgtgtctgcacatgtgcataagtgtgtatgcggggtg

ttcagagccctggggcactgagcatttagagatttagagcttaggtacaacaatgtccct

gaataaagctctgacccacgtgccccagtcttggttggattcatgttaaagtcagagaag

tgtagtccgacaaccaacagtaatttcaaaagttattcgaggtcttcttcatctgcacct

ctcaaacactagtaaagggatgcagatccaccgtaaaacacctgaacatgcctccaatct

tcaatcacctatctctttaaccaatgtaagcatctattgtacctctttctttcttggaaa

atctccttaaaactccttctccagtcctatccatgcccctttggatcttgtagtcttttg

caccctccgccccctttcttcctccaggctgagactcttacccagtttctcttgttccgt

cctatgcaatccccgccccctcaaagtgtacctacttccagaagcacctggggacaggg

gtggggtgagtcctgggggtggtgctgatgagagaatccgggctggtaaaccaaacctca

tacatacacacatagggtcatgatggtcccctcacatttcccgattctccatatggtgga

ggaaaccaggtgttcaagggacttgctaaccagcctccttcgtgtggagccctaggcagg

cacgtttgaggcccaggaaatcctcgcccagcgggtggccggggctctggagggagtgtg

gcaggtggcagggcggtcattgcacagatacacatttgcaagagaccactagggcctgcg

aggggggggggcgagggaggggcccccccgggaagcctcactcggggaggcccaggctccc

ctgactgccccggcggcgtctcacctgagatcagctcagcgtctttgcagccggtggctc

cgaacccccacggagggccgctaggaccccggcttccaggtgaggaagctgcggcccagg

gaggcgggaggtggcgggcggggcggggcggggcggggccgcaggggaggctcccggcag

ggagggcgcgggacccccgcccaggcgcccctcgtcccgcgcaggttccggcagctgctg
                                                F  R  Q  L  L
gccgagcgcgagcccgaggtgcacgaggtgtcgcggctcttccgctcggtgctgcaggag
 A  E  R  E  P  E  V  H  E  V  S  R  L  F  R  S  V  L  Q  E
gtcctggagcgcgcgcagcgcg}
 V L E R A Q R          3'
                        9875470
```

**Supplementary Fig. S13**. Upper panel showing the sequence alignment of 115 base pairs of the contig bc_85943 to chr7:9875357-9875470 with 4 mismatches and 1 gap and 1498 unaligned base pairs at the 3' end of the contig. Lower panel demonstrating the presence of partial exon 4 of RD3 splice variant within the 1498 unaligned base pairs missing from the reference (Kukekova et al. 2009). The sequence in {} represents the missing sequence from the reference which contains partial sequence of exon 4 of RD3 splice variant. The underlined nucleotides represent the complete sequence of the contig bc_85943 while the light green nucleotides represent the non-coding sequence/intron and dark green nucleotides represent the coding sequence.

**Supplementary Notes.**

Sequence alignment of contig bc_c156488 to FAM161A1 at chr10:61814401-61816040 in reverse orientation to the reference. The nucleotides in red indicate the stop codons in both the contig and reference. A 445 bp insertion in the reference at chr10: 61814980-61815425 terminating the protein product with stop codon (TAA) at 61815429-61815431.

5’

```
bc_rc156488 1         tttagttttgccctttggggactttaggtanatgagtcattcattctat    50
                      |||||||||||||||||||||||||||||| |||||||||||||||||||
Subject 61816040      TTTAGTTTTGCCCTTTTGGGGACTTTAGGTAAATGAGTCATTCATTCTAT    61815991

bc_rc156488 51        gtgtattctcttgtcttttgcttcttttgcttcgtactgtgttcatgaga   100
                      ||||||||||||||||||||||||||||||||.|||||||||||||||||
Subject 61815990      GTGTATTCTCTTGTCTTTTGCTTCTTTTGCTTAGTACTGTGTTCATGAGA   61815941

bc_rc156488 101       tccatccatactgtcgtgtacgatagaattcatttattgtcattgcttta   150
                      ||||||||||||||||||||||||||||||||||||||||||||||||||
Subject 61815940      TCCATCCATACTGTCGTGTACGATAGAATTCATTTATTGTCATTGCTTTA   61815891

bc_rc156488 151       ttttactccgttgtgtgaatagaccattatttatcaatcctattgttgag   200
                      ||||||||||||||||||||||||||||||||||||||||||||||||||
subject 61815890      TTTTACTCCGTTGTGTGAATAGACCATTATTTATCAATCCTATTGTTGAG   61815841

bc_rc156488 201       agatatttgggttgtttccagtttggggttgttaaaagtagtactttcct   250
                      ||||||||||||||||||||||||||||||||||||||||||||||||||
subject 61815840      AGATATTTGGGTTGTTTCCAGTTTGGGGTTGTTAAAAGTAGTACTTTCCT   61815791

bc_rc156488 251       gagcgtttttgtacacgtcttctggggtacatatgtaggagttcctctgg   300
                      ||||||||||||||||||||||||||||||||||||||||||||||||||
subject 61815790      GAGCGTTTTTGTACACGTCTTCTGGGGTACATATGTAGGAGTTCCTCTGG   61815741

bc_rc156488 301       gtatatgctggggagtgggattgctggtcagaaggtttgcttactttgag   350
                      ||||||||||||||||||||||||||||||||||||||||||||||||||
subject 61815740      GTATATGCTGGGGAGTGGGATTGCTGGTCAGAAGGTTTGCTTACTTTGAG   61815691

bc_rc156488 351       tcaactgtgattattagatttaatttctaatactggagcttttaacccat   400
                      ||||||||||||||||||||||||||||||||||||||||||||||||||
subject 61815690      TCAACTGTGATTATTAGATTTAATTTCTAATACTGGAGCTTTTAACCCAT   61815641

bc_rc156488 401       acatttttaatgaaaatttaaagtcttttgttctcctctaaaaaggtttg   450
                      ||||||||||||||||||||||||||||||||||||||||||||||||||
subject 61815640      ACATTTTTAATGAAAATTTAAAGTCTTTTGTTCTCCTCTAAAAAGGTTTG   61815591

bc_rc156488 451       tctttcctatagaaaaatgcaagaatggcagcagaaaagcgttattctaa   500
                      ||||||||||||||||||||||||||||||||||||||||||||||||||
subject 61815590      TCTTTCCTATAGAAAAATGCAAGAATGGCAGCAGAAAAGCGTTATTCTAA   61815551

bc_rc156488 501       caccctaaaagcattaggactatctgatgagtttgtttcaaagaaaggcc   550
                      ||||||||||||||||||||||||||||||||||||||||||||||||||
subject 61815550      CACCCTAAAAGCATTAGGACTATCTGATGAGTTTGTTTCAAAGAAAGGCC   61815491

bc_rc156488 551       aaaatggaaaagtatttgagtacttcaacaatcaagagatgaagaatttc   600
                      |||.|||||||||||||||||||||||.||||||||||||||||||||||
subject 61815490      AAAGTGGAAAAGTATTTGAGTACTTCAGCAATCAAGAGATGAAGAATTTC   61815441

bc_rc156488 601       actgaagataaagaa-----------------------------------   615
                      |||||||||||||||
subject 61815440      ACTGAAGATAAAGAAAGGTAACATATATAGGACATCTAAATGGTTTACCT   61815391

bc_rc156488 616       --------------------------------------------------   615

subject 61815390      TTGGAAAAAATTCTTACTTGCTGGAAAGAATAAATTCTGTCCTATATCTTT  61815341

bc_rc156488 616       --------------------------------------------------   615
```

```
subject 61815340      AAAAGATCTTTTAACCTAACACAATACATGTGAACTGTTGTAAAACATTG    61815291


bc_rc156488 616       --------------------------------------------------    615

subject 61815290      GTATTTTAACACTTGGCACATTGAAAATATTATGTGGTGATGAAGTATCT    61815241

bc_rc156488 616       --------------------------------------------------    615

subject 61815240      TGTTTTTTAAAGCAGAATAATTTAAATTTCTGACTGACATAAATGATGAA    61815191

bc_rc156488 616       --------------------------------------------------    615

subject 61815190      AATAATAAGATGCTTTCAAATGAAGCTTACAGAGCCAAACAAGGTTTGAA    61815141

bc_rc156488 616       --------------------------------------------------    615

subject 61815140      ATAGATTAGTTCTCAATTAGCACTTTTATAGAGCTGTCTTGGTAAATCAG    61815091

bc_rc156488 616       --------------------------------------------------    615

subject 61815090      AGCTCATGCCCTTGAGGAAGACTGACTCCATGATGATATACGTTGCAAAG    61815041

bc_rc156488 616       --------------------------------------------------    615

subject 61815040      CGTTGGTTGACCTTTAGACCAAATCATTTTTCCTTTGAAAGATTGATAAA    61814991

bc_rc156488 616       -----------acctttgacgaagaagaaaaaatagaagaaagagcgaat    654
                                 |.||||||.|||||||||||..|||||||||||||||.|
subject 61814990      TTTCTTCCTCCAGCTTTGATGAAGAAGAAAATGTAGAAGAAAGAGCGAGT    61814941

bc_rc156488 655       ggggaagaaaatcattttattgatactgaacagccaggattcttacaaga    704
                      |||||||||||||||||||||||||| |||||||||||||||||||||| 
subject 61814940      GGGGAAGAAAATCATTTTATTGATAC-GAACAGCCAGGATTCTTACAAG-    61814893

bc_rc156488 705       aaaaaagaggaagctgatgaagagagtggagaagagaaatctgttaagga    754
                      .|||||||||||.|||||||||||||||||||||||||||||||||||||
subject 61814892      GAAAAAGAGGAAACTGATGAAGAGAGTGGAGAAGAGAAATCTGTTAAGGA    61814843

bc_rc156488 755       gtaagagggaatcagcaggatgtctcctgtctgtgccngngctgtcgatg    804
                      ||||||||||||.|||||||||||||||||||||||| | ||||||||||
subject 61814842      GTAAGAGGGAATGAGCAGGATGTCTCCTGTCTGTGCCCGCGCTGTCGATG    61814793

bc_rc156488 805       gcagcccctggcattagcatccgtgcttatggtgggaacagcatcagtga    854
                      |||||||||||||||||||||||||||||.||||||||||||||||||||
subject 61814792      GCAGCCCCTGGCATTAGCATCCGTGCTTACGGTGGGAACAGCATCAGTGA    61814743

bc_rc156488 855       gagccctccaacctgtacaaggctcttaagcaaagtcatctgtagcctgg    904
                      |||||||||||||||||||||||||||.||||||||||||||||||||||
subject 61814742      GAGCCCTCCAACCTGTACAAGGCTCTTGAGCAAAGTCATCTGTAGCCTGG    61814693

bc_rc156488 905       aaaggccaaatccaaccaaaaggttgttgggtcagttggagaaaggcttt    954
                      |||||||||||||||||.||..|||||||||||||||||||.||||||||
subject 61814692      AAAGGCCAAATCCAACCGAATTGTTGTTGGGTCAGTTGGAGTAAGGCTTT    61814643

bc_rc156488 955       gcttgttcaggttttttaatcactacatatggtttgcaactttgatctta    1004
                      ||.||||||||||||||||||||||..||||.|||||||||||||.||||
subject 61814642      GCCTGTTCAGGTTTTTTAATCACTATGTATGATTTGCAACTTTGACCTTA    61814593

bc_rc156488 1005      cttgtatttttaaaaaagcatttgagaatcatagctg---tcgtaaattg    1051
                      |||||||||||||||||||||||||||||||||||||   ||.||||.||
subject 61814592      CTTGTATTTTTAAAAAAGCATTTGAGAATCATAGCTGTCATCATAAACTG    61814543

bc_rc156488 1052      attaattgttaacatatttgctacttccttatttgaatgggaaaaaatgg    1101
                      ||||.||.|.||||||||||||||||||||||||||||||||||||||||
subject 61814542      ATTAGTTCTGAACATATTTGCTACTTCCTTATTTGAATGGGAAAAAATGG    61814493
```

```
bc_rc156488 1102        cttggcattgagagcagagaaaagtactggcttttggtgatttgaaaaaa   1151
                        ||||||||||||||||||.|.|||||||||||||||||||||||||||||
subject 61814492        CTTGGCATTGAGAGCAGAAAGAAGTACTGGCTTTTGGTGATTTGAAAAAA   61814443

bc_rc156488 1152        tgtttctgggaagtctgaatcaataaatattttaaaattataaaaaataa   1201
                        |||||.||||||||||||||||||||||||||||||||||||
subject 61814442        TGTTTGTGGGAAGTCTGAATCAATAAATATTTTAAAATTATA--------   61814401

bc_rc156488 1202        caattggagatatatgcttccttgttcatagtggaaattaagcacctctg   1251

subject                 --------------------------------------------------   61814341

bc_rc156488 1252        ccccaatcataggttaaaattatttcttttcttccttattatgccaatgt   1301

subject                 --------------------------------------------------   61814191

bc_rc156488 1302        gggtcacatcgtacccactctggtcacttcagccatggtccttgaacaaa   1351

subject                 --------------------------------------------------   61814141

bc_rc156488 1352        ttaccatccattccacccctcctctataatatatagttatattataatta   1401

subject                 --------------------------------------------------   1640

bc_rc156488 1402        taatatataattccccattgctatactataaggtgagtggaagctagggt   1451

subject                 --------------------------------------------------   1640
```

3’

Sequence translation of the contig bc_c156488. The nucleotides in green represent the CDS sequence of exon 6 in FAM161A in the contig that codes for 188 aa where ‘^’ marks the position of stop codon in the reference sequence after 51 aa. The underlined CDS sequence (82 aa) represents similarity to protein FAM161A isoform X3 (XP_005626198.1) of the mRNA transcript XM_005626141.3.

```
aaaaatgcaagaatggcagcagaaaagcgttattctaacaccctaaaagcattaggacta
 K  N  A  R  M  A  A  E  K  R  Y  S  N  T  L  K  A  L  G  L
tctgatgagtttgtttcaaagaaaggccaaaatggaaaagtatttgagtacttcaacaat
 S  D  E  F  V  S  K  K  G  Q  N  G  K  V  F  E  Y  F  N  N
Caagagatgaagaatttcactgaagataaagaa^acctttgacgaagaagaaaaaatagaa
 Q  E  M  K  N  F  T  E  D  K  E   T  F  D  E  E  E  K  I  E
gaaagagcgaatggggaagaaaatcattttattgatactgaacagccaggattcttacaa
 E  R  A  N  G  E  E  N  H  F  I  D  T  E  Q  P  G  F  L  Q
gaaaaaaagaggaagctgatgaagagagtggagaagagaaatctgttaaggagtaagagg
 E  K  K  R  K  L  M  K  R  V  E  K  R  N  L  L  R  S  K  R
gaatcagcaggatgtctcctgtctgtgccngngctgtcgatggcagcccctggcattagc
 E  S  A  G  C  L  L  S  V  X  X  L  S  M  A  A  P  G  I  S
atccgtgcttatggtgggaacagcatcagtgagagccctccaacctgtacaaggctctta
 I  R  A  Y  G  G  N  S  I  S  E  S  P  P  T  C  T  R  L  L
agcaaagtcatctgtagcctggaaaggccaaatccaaccaaaaggttgttgggtcagttg
 S  K  V  I  C  S  L  E  R  P  N  P  T  K  R  L  L  G  Q  L
gagaaaggctttgcttgttcaggtttttttaatcactacatatggtttgcaactttgatct
 E  K  G  F  A  C  S  G  F  L  I  T  T  Y  G  L  Q  L  -  S
tacttgtatttttaaaaaagcatttgagaatcatagctgtcgtaaattgattaattgtta

acatatttgctacttccttatttgaatgggaaaaaatggcttggcattgagagcagagaa

aagtactggcttttggtgatttgaaaaaatgtttctgggaagtctgaatcaataaatatt
```

ttaaaattataaaaaataacaattggagatatatgcttccttgttcatagtggaaattaa

gcacctctgccccaatcataggttaaaattatttcttttcttccttattatgccaatgtg

ggtcacatcgtacccactctggtcacttcagccatggtccttgaacaaattaccatccat

tccacccctcctctataatatatagttatattataattataatatataattccccattgc

tatactataaggtgagtggaagctagggt
